# Arrhythmia Detection in Atrioventricular, Single-Lead, Floating Atrial Dipole ICD Systems Compared with Conventional Single- and Dual-Chamber Defibrillators

**DOI:** 10.3390/jcdd11120386

**Published:** 2024-12-01

**Authors:** Flora Diana Gausz, Kom Nangob Manuela Lena, Paul Emmanuel Gedeon, Marton Miklos, Attila Benak, Gabor Bencsik, Attila Makai, Dora Kranyak, Rita Beata Gagyi, Robert Pap, Laszlo Saghy, Tamas Szili-Torok, Mate Vamos

**Affiliations:** 1 Cardiac Electrophysiology Division, Cardiology Center, Department of Internal Medicine, University of Szeged, 6725 Szeged, Hungary; 2 Department of Oncotherapy, University of Szeged, 6720 Szeged, Hungary; 3 Neurosurgery, Queen Elizabeth University Hospital, G51 4TF Glasgow, UK

**Keywords:** ICD, implantable cardioverter defibrillator, floating atrial sensing dipole, VDD, tachycardia discrimination, atrial arrythmia detection

## Abstract

Background: An atrioventricular defibrillator system with a floating atrial dipole (VDD ICD) can provide atrial sensing by a single lead. Our aim was to compare the arrhythmia detection efficacy of VDD ICDs with conventional single- (VVI) and dual-chamber (DDD) defibrillators. Methods: Data from consecutive patients undergoing ICD implantation were retrospectively analyzed. The primary endpoint was the incidence of device-detected, new-onset atrial arrhythmias, while secondary endpoints were sensing parameters, complication rates, incidence of appropriate/inappropriate ICD therapy, arrhythmic/heart failure-related hospitalizations, and all-cause mortality. Results: A total of 256 patients (mean age 64 ± 12 years, male 75%, primary prophylaxis 28%, mean follow-up 3.7 ± 2.4 years) were included (VVI: 93, VDD: 94, DDD: 69). Atrial arrhythmia episodes were detected more frequently by VDD systems compared to VVI ICDs (aHR 7.087; 95% CI 2.371–21.183; *p* < 0.001), and at a rate similar to that of DDD ICDs (aHR 1.781; 95% CI 0.737–4.301; *p* = 0.200). The rate of inappropriate shocks was not different among the three ICD systems. Conclusion: VDD devices revealed an advantage in atrial arrhythmia detection compared to VVI ICDs and were non-inferior to DDD systems. Their main indication may be closer monitoring in high-risk patients with atrial arrhythmias to help therapy optimization and not the improvement of tachycardia discrimination.

## 1. Introduction

Implantable cardioverter defibrillators (ICD) are known to have an essential role in the prevention of sudden cardiac death by appropriate detection and early termination of malignant ventricular arrhythmias [1]. Conventional transvenous single-lead implantable cardioverter defibrillators (VVI ICD) offer proper ventricular sensing and pacing with a single right ventricular lead and are capable of direct ventricular arrhythmia detection. VVI systems are limited in detecting atrial tachyarrhythmias (TA), since this is only possible in an indirect way by analyzing the irregular ventricular signals (supplemented with morphology discriminators in modern devices) [2].

On the other hand, conventional dual-chamber (DDD) ICD systems are able to sense and pace in the ventricle as well as in the atrium provided by an implanted lead in the right atrium that can process direct atrial signals. With an early detection of atrial high-rate episodes (AHREs), subclinical atrial fibrillation and/ or atrial flutter can be detected, leading to adequate oral anticoagulation. Furthermore, early steps in rhythm control can be initiated too [3,4,5]. By proper atrial sensing, DDD ICD devices are more effective in atrial TA detection compared with conventional VVI devices [6]. However, the implantation of more leads lengthens the procedure duration and may impose the additional risk of early and late complications [7,8,9,10,11].

VDD implantable cardioverter defibrillator systems (also known as Diagnostic eXtension (DX) ICD systems) are equipped with a floating atrial sensing dipole providing high-quality atrial sensing [6,7]. Large studies, like SENSE and THINGS, showed superiority to VVI ICDs and non-inferiority to DDD-ICDs in case of novel atrial tachycardia detection [6,12]. Moreover, VDD ICD devices can provide closer and reliable remote patient care by the combination with a remote monitoring system [4,13].

VDD ICD systems can be programmed to discriminate between ventricular and supraventricular arrhythmias with both single- and dual-chamber discriminators [7,14]. Notably, there are controversial results regarding the superiority of dual vs. single-chamber discriminators in the literature [8,15,16,17,18,19].

In our retrospective, single-center study, we aimed to compare TA detection and discrimination in VDD ICD devices with conventional single- and dual-chamber ICD systems. We wanted to verify the application of VDD devices in our center by examining the efficacy of atrial TA detection supposing superiority in comparison with VVI systems. We also gathered information about TA discrimination capabilities of VDD devices with regard to controversial statements of this topic in the literature. Finally, we also studied long-term clinical outcomes (i.e., hospitalization due to arrhythmic and heart failure events and all-cause mortality), knowing that only sparse literature data are given on this topic.

## 2. Materials and Methods

Data from consecutive patients undergoing ICD implantation from all manufacturers with standard indications between 2009 and 2023 at the Cardiology Center of the University of Szeged were retrospectively collected. Baseline demographics, main comorbidities, data regarding ICD indication, and key information regarding the implanted devices were analyzed. We scanned the main cardiovascular background involving ischemic/non-ischemic etiology, previously diagnosed atrial fibrillation or atrial flutter, left ventricular ejection fraction (LVEF), known hypertension, dyslipidemia, and diabetes mellitus. We collected data regarding baseline laboratory parameters, medical therapy, and ECG parameters before ICD implantation. Patients with biventricular cardioverter defibrillators were excluded. The study was approved by the Institutional Review Board (IRB) of the University of Szeged (No. 4870) and it conforms to the ethical guidelines of the Declaration of Helsinki.

### 2.1. Study Endpoints

Based on the type of the implanted device, patients were divided into 3 groups: VVI ICD, VDD ICD, and DDD ICD. The primary outcome of this study was the incidence of the first device detected atrial arrhythmia. Before statistical analysis of this parameter, we excluded patients with known permanent atrial fibrillation. Then, we collected episodes of new-onset clinical or subclinical atrial fibrillation (or atrial flutter). Any AHREs longer than 1 min were considered. The secondary endpoints were atrial (at 6 months after implantation and at the end of the follow-up) and ventricular sensing parameters and pacing percentage (at 6 months after implantation), incidence of appropriate/inappropriate ICD therapy, incidence of hospitalization due to arrhythmic and heart failure events, and all-cause mortality. Short-term and long-term complications defined as pneumothorax, bleeding, thrombosis, lead- or device-related complications, repeated surgery, and CIED-related infections were also analyzed. Arrhythmia-related hospitalizations involved acute admission due to arrhythmic events, device-related problems, and hospital admissions aiming at rhythm control of atrial fibrillation or flutter (i.e., electrical cardioversion (ECV), catheter ablation of atrial fibrillation/flutter).

### 2.2. Statistical Analysis

Data were collected in an Excel document (Microsoft, Redmond, WA, USA) and statistical analyses were performed with SPSS Statistics (version 27, IBM, Armonk, NY, USA). Continuous variables are expressed as mean ± standard deviation (SD) and categorical variables as numbers (percentages).

To compare baseline clinical characteristics, baseline medication, and certain clinical outcomes like sensing or pacing parameters (at 6 months) and complication rates, we used one-way analysis of variance (ANOVA) analysis for continuous variables (in case of non-normal distribution evaluated by Kruskal–Wallis test) and Chi-squared test for categorical variables. When we compared only 2 groups (subgroup analysis and atrial sensing at 6 months and at the end of the follow-up) we used the independent samples *t*-test for continuous parameters (in the case of non-normal distribution, we performed Mann–Whitney U test) and Chi-squared test for categorical variables.

Atrial arrhythmia detection, appropriate and inappropriate ICD therapy, cardiac arrhythmic hospitalization, heart-failure related hospitalization, and all-cause mortality were compared between the patient groups using a time-to-event analysis calculating hazard ratios (HR) along with a 95% confidence interval (CI). Statistical significance was determined as a *p*-value ≤ 0.05. Furthermore, we expanded the statistical comparison with a multivariate model by including all predictor variables that we considered to have an impact on the outcome and resulted in a significance level of *p*-value ≤ 0.1. Predictor variables involved in the multivariate model depending on the significance level of univariate COX-regression were the following: age, gender, primary prophylaxis, ischemic etiology, previously diagnosed atrial fibrillation, hypertonia, dyslipidemia, diabetes mellitus, stroke/transient ischemic attack (TIA), bradypacing indication, baseline LVEF, QRS width, heart rate, baseline creatinine and hemoglobin levels, remote monitoring, antiplatelet therapy, anticoagulation, therapy with beta-blocker, angiotensin-converting-enzyme inhibitor (ACEI)/angiotensin II receptor blocker (ARB)/angiotensin receptor-neprilysin inhibitor (ARNI), diuretics, calcium channel blocker, mineralocorticoid receptor antagonist, statin, amiodarone, and digitalis glycosides. ICD type was involved in every multivariate calculation irrespectively of the significance level of the univariate analysis of this parameter.

## 3. Results

### 3.1. Baseline Clinical Characteristics and Medical Therapy

The mean age at device implantation was 64 years; 75% of the study population was male, 61% had ischemic etiology, and the indication for ICD implantation was primary prophylactic in 28% of cases. A total of 93 patients received a VVI, 94 received a VDD, and 69 received a DDD ICD system.

Comparison of comorbidities and baseline characteristics between the study groups are summarized in Table 1. A difference was only detected in the prevalence of dyslipidemia and in mean LVEF (Table 1 and Table S1 in Supplementary Materials). Patients implanted with a DDD system had a lower prevalence of dyslipidemia and a higher mean LVEF.

The baseline ECG characteristics showed differences in QRS width between the DDD group and the other groups. There was a tendency for bradycardia in DDD group compared with the other two groups. Anti-bradycardia pacing indication was significantly higher in the DDD group, while the vast majority of patients in the VVI and VDD groups did not have conduction system disturbances (Table 1 and Table S1). Since Biotronik is the only manufacturer of the DX ICD systems, 98% of patients in VDD ICD group were implanted with a Biotronik generator; in the remaining two cases, a VDD ICD lead was used with a St. Jude Medical (SJM) or Sorin generator. In the VVI and DDD ICD groups, generators from various manufacturers (i.e., Biotronik, Boston Scientific, Medtronic, and SJM) were used (Table 1).

In the baseline laboratory parameters, we detected a significant difference in hemoglobin levels, which were the highest in the VDD group (Table 1 and Table S1).

A remote monitoring system was most frequently applied in the VDD group involving 51% of DX ICD recipients (Table 1 and Table S1).

Comparing baseline medical therapy, more beta-blockers and mineralocorticoid receptor antagonists were used in the VVI and VDD groups (Table 2 and Table S2).

### 3.2. Clinical Outcomes

#### 3.2.1. Device Detected Atrial Arrhythmias

**Figure 1:**
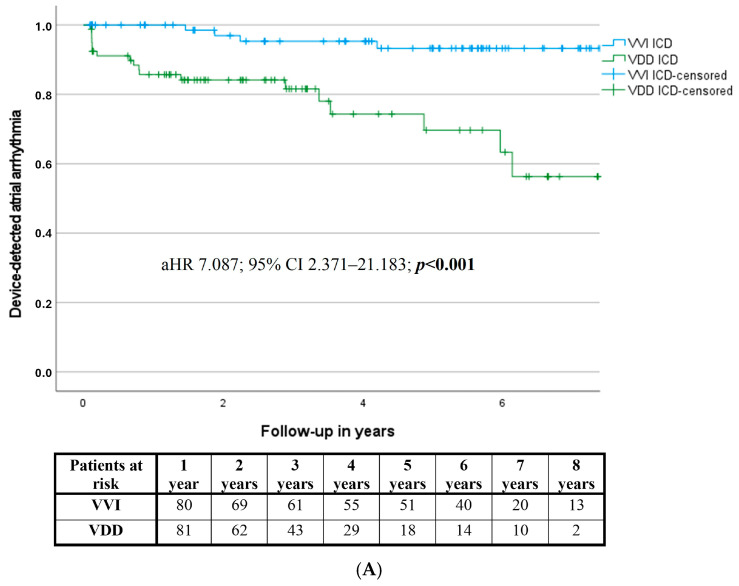

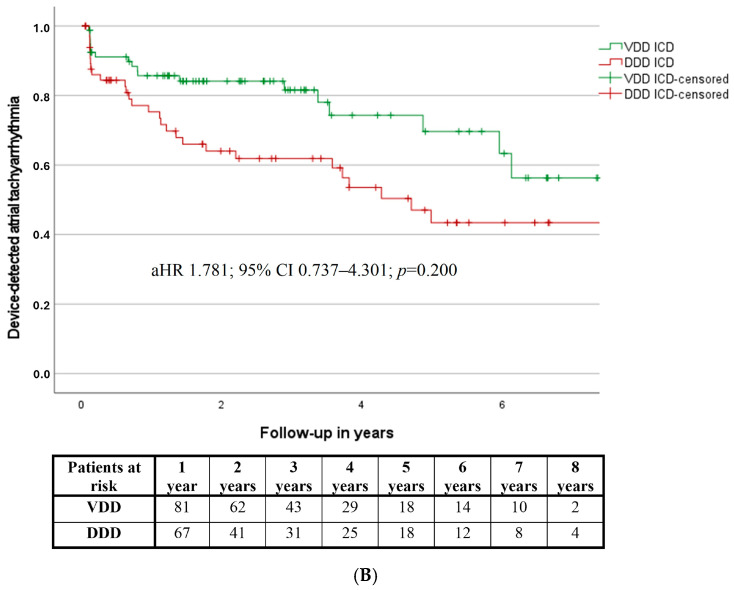
(**A**) Time to first device-detected atrial arrhythmia—VVI vs. VDD. (**B**) Time to first device detected atrial arrhythmia—VDD vs. DDD. aHR: adjusted hazard ratio.

During the mean follow-up of 3.7 years, the incidence of device-detected atrial arrhythmia episodes was significantly higher in patients implanted with a VDD ICD system compared to the conventional single-lead ICD recipients (HR 6.506; 95% CI 2.176–19.446; *p* = 0.001; adjusted hazard ratio (aHR) 7.087; 95% CI 2.371–21.183; *p* < 0.001). However, univariate comparison of VDD vs. DDD systems revealed a higher rate of atrial arrhythmia detection in the DDD group; after adjustment for the clinically and statistically relevant confounders, no significant difference between the two devices was identified (aHR 1.781; 95% CI 0.737–4.301; *p* = 0.200) (Table 3, Tables S3 and S4, Figure 1A,B). Notably, the distribution of the different atrial arrhythmia episodes (paroxysmal atrial fibrillation, persistent atrial fibrillation, and regular atrial arrhythmia) within device detected atrial arrhythmias was similar among the three groups (*p* = 0.609) (Table S5).

#### 3.2.2. Sensing and Pacing Parameters

**Figure 2:**
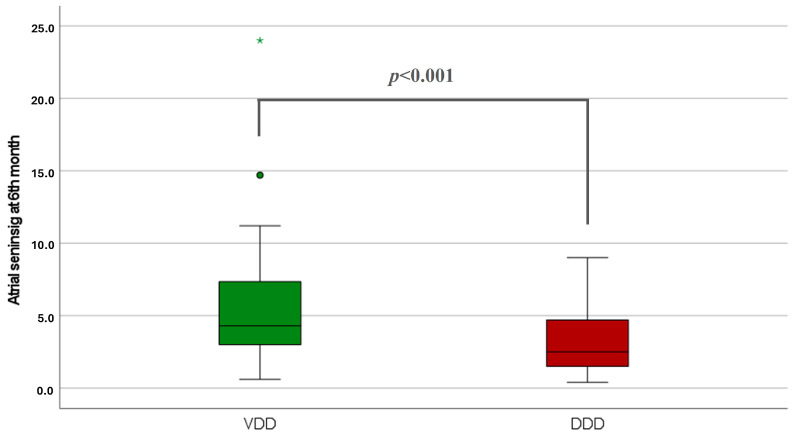
Atrial sensing in VDD vs. DDD ICDs at 6th month. * and 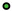: it represents an outliner value.

VDD ICD devices provided higher atrial sensing amplitude at the 6th month compared with conventional DDD ICDs (5.3 ± 3.7 vs. 3.1 ± 2.1 mV; *p* < 0.001) (Table 4, Figure 2). Higher atrial sensing parameters were also detected in the VDD ICD group at the end of the follow-up (4.2 ± 3.2 vs. 2.7 ± 1.8 mV; *p* = 0.009) (Table 4). There was no significant difference in ventricular sensing among the three groups (VVI 12.8 ± 4.8 vs. VDD 14.0 ± 6.0 vs. DDD 13.2 ± 5.7 mV; *p* = 0.313) (Table 4, Figure S1). The mean atrial pacing percentage at 6 months was 23% in DDD devices. A significant difference in ventricular pacing percentage was measured with highest value in the DDD ICD group (VVI 2.2 ± 7.0 vs. VDD 2.8 ± 14.4 vs. DDD 33.6 ± 41.9%; *p* < 0.001) (Table 4).

#### 3.2.3. Complications

The prevalence of complications was the highest in the DDD group (20%), 13% with the VDD systems group, and 8% in patients implanted with a VVI ICD (Table 4). The subgroup analysis detected a significant difference only at the VVI vs. DDD comparison, but not between the VVI and VDD or VDD and DDD ICD groups (Table 4 and Table S6). The details of the distribution of complications can be found in Table S7.

#### 3.2.4. Tachyarrhythmia Discrimination—Appropriate and Inappropriate ICD Therapy

Comparison of VDD systems with VVI devices revealed no significant difference in the incidence of appropriate ICD therapies (aHR 0.983; 95% CI 0.641–1.508; *p* = 0.937); moreover, we did not identify any difference in the rate of inappropriate therapy (aHR 0.742; 95% CI 0.313–1.757; *p* = 0.497) (Table 3, Tables S8 and S10, Figure S2A,C). The comparison of the VDD vs. DDD systems also showed equality both for appropriate (aHR 0.651; 95% CI 0.371–1.142; *p* = 0.135) and inappropriate (aHR 0.618; 95% CI 0.203–1.878; *p* = 0.396) therapy delivery (Table 3, Tables S9 and S11, Figure S2B,D).

#### 3.2.5. Arrhythmia and Heart Failure Hospitalization

A significant difference was identified regarding hospitalization of arrhythmic cause between VVI and VDD ICD groups (aHR 1.706; 95% CI 1.043–2.792; *p* = 0.033) (Table 3 and Table S12, Figure S3A). However, there was no difference between the VDD and DDD groups for arrhythmic hospitalization (aHR 0.700; 95% CI 0.365–1.341; *p* = 0.282) (Table 3 and Table S13, Figure S3B). The detailed distribution of arrhythmia-related hospitalization events is described in Table S14. The risk of heart failure-related hospitalizations was similar across all groups (aHR VVI vs. VDD 1.628; 95% CI 0.619–4.279; *p* = 0.323 and aHR VDD vs. DDD 0.949; 95% CI 0.301–2.991; *p* = 0.928) (Table 3, Tables S15 and S16, Figure S4A,B).

#### 3.2.6. All-Cause Mortality

**Figure 3:**
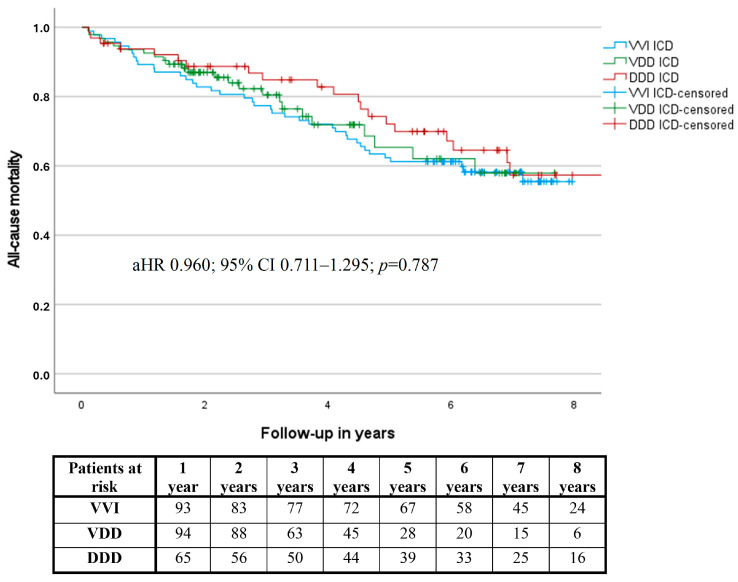
All-cause mortality in all groups.

There was no significant difference regarding all-cause mortality among the three groups (aHR 0.960; 95% CI 0.711–1.295; *p* = 0.787) (Table 3 and Table S17, Figure 3).

## 4. Discussion

### 4.1. Main Findings

In this retrospective, single-center study, we aimed to compare TA detection and discrimination efficacy of VDD ICDs with conventional single- (VVI) and dual-lead (DDD) systems. Our results for device-detected, new-onset atrial TA demonstrated clear advantages for the VDD ICD group compared with VVI systems. This confirms the benefit of direct atrial sensing provided by the floating atrial sensing dipole of the DX ICD. Moreover, the adjusted comparison with DDD ICD systems showed the non-inferiority of VDD ICDs in atrial TA detection. In TA discrimination, no significant difference among the three types of ICD were detected. Trends towards more complications with DDD systems should be also highlighted. In this group, the most frequent complications were infection- (six cases) and lead-related (five cases) (Table S7). The current study compared VDD ICD devices with conventional single-chamber and dual-chamber devices within a single investigation, using data from the same center, whereas previous studies focused only on comparisons of VVI vs. VDD or VDD vs. DDD devices. Detailed data were provided on the distribution of different types of device-detected atrial arrhythmias and on specific causes of arrhythmia-related hospitalization events. Moreover, to the best of our knowledge, this is the first study demonstrating the superiority of atrial sensing by DX ICD devices compared to dual-lead ICDs.

### 4.2. Early Detection of Atrial Arrhythmias

Our data harmonize with those found in the international literature, emphasizing the advantages of VDD systems in novel atrial TA detection. The results of a progressive, multi-center study based on the THINGS registry showed the superiority of VDD ICD systems, with almost four times efficacy in atrial arrhythmia detection compared with conventional VVI devices [12]. The SENSE trial studied AHRE detection in VDD devices compared with both conventional single-chamber and dual-chamber devices and revealed the superiority in subclinical atrial fibrillation detection of VDD devices compared with VVI systems and non-inferiority in comparison with DDD devices [6]. Furthermore, the MATRIX registry investigated VDD systems’ ability in AHRE identification via remote monitoring and demonstrated 99.7% accuracy in the detection of AHRE episodes lasting ≥ 1 h [4]. The reliability of atrial sensing by the DX dipole was also confirmed by previous reports [4,6,20]. In our measurements, atrial sensing was significantly higher for DX ICD devices compared to dual-lead ICDs, and we are first to report the superiority of VDD ICD regarding this parameter. Highly reliable atrial sensing in VDD ICD devices is established by a four-fold amplifier and a wider bandpass filter [7]. With trustable and early atrial fibrillation detection, adequate oral anticoagulation therapy can be initiated with consideration of early rhythm control, resulting in a decrease in adverse consequences of atrial fibrillation (increased mortality, stroke risk, vascular dementia, heart failure, hospitalization, impaired quality of life, etc.) [4,5,6,21]. Further, a recent, nationwide, prospective survey revealed an elevated risk of stroke as most important influencing factor for the implanters to select a DX ICD instead of a conventional single-chamber or dual-chamber ICD systems [22]. The first prospective, multicenter, randomized-controlled trail regarding subclinical atrial fibrillation detection in VDD ICD devices compared to VVI ICD systems was the Dx-AF study. Although the Dx-AF study demonstrated no significant difference in the efficacy of atrial fibrillation/flutter detection between the two groups (evaluating detection by the device, ECG, or ECG monitoring), the results revealed a borderline significant difference in the efficacy of device-detected atrial arrhythmias favoring VDD devices [23]. The more powerful statistical difference between VVI and VDD devices regarding device-detected atrial arrhythmias in our results could be explained by the different cut-off for the AHRE episodes. While, in our study, the minimal limit of AHRE episodes was 1 min, the Dx-AF study considered AHRE episodes lasting at least 6 min. In the literature, the most common limit for AHRE detection is 6 min since the ASSERT study [24]. However, shorter episodes of AHRE can be associated with thromboembolic events as well [25]. In our study, we aimed to choose a properly sensitive duration limit for AHRE episodes, resulting in a defined limit of 1 min.

The administration of oral anticoagulants in patients with atrial high-rate episodes has become a compelling topic recently, given the results of the NOAH-AFNET and ARTESiA trials. These trials investigated whether direct oral anticoagulants reduced the risk of stroke or systemic embolism compared to control (placebo or aspirin). While the ARTESiA trial showed a statistically significant reduction in stroke and systemic embolism with apixaban, the NOAH-AFNET trial (applying edoxaban) did not demonstrate a significant benefit for the primary composite endpoint of cardiovascular death, stroke, or embolic events, including systemic embolism, myocardial infarction, and pulmonary embolism [26,27]. A meta-analysis of both trials confirmed a consistent reduction in stroke and systemic embolism (35%) with edoxaban or apixaban compared to control/aspirin, but also a consistent increase in major bleeding events. Based on these studies, there is a clear association between device-detected atrial fibrillation and an increased risk of ischemic stroke or systemic embolism. It is also important to emphasize the role of individual risk profiles and shared decision-making to find the appropriate solution for patients with device-detected atrial fibrillation regarding the administration of oral anticoagulants [5,28].

### 4.3. Role of the Atrial Signal in Tachycardia Discrimination

The analysis of atrial signals is not only beneficial in atrial arrhythmia detection but may also be useful for TA discrimination. By atrial sensing, VDD systems can be programmed to differentiate between ventricular and supraventricular arrhythmias by dual-chamber discriminators. There are controversial results in the literature regarding the advantage of dual-chamber discriminators over single-chamber algorithms. The availability of atrial signal detection initiates the opportunity to compare the atrial vs. ventricular rate, analyze atrial electrogram (EGM), and gather information regarding atrioventricular (AV)-association [14,29]. In some smaller studies, dual-chamber discriminators showed superiority compared to single-chamber discriminator algorithms [15,16,30]. Kurt et al. compared VDD ICDs and conventional VVI devices regarding inappropriate ICD therapy rate and their results showed the superiority of the VDD system [8]. However, other studies conclude that newly developed, modern, and accurate morphology discriminators applied in single-chamber systems seem to make them just as reliable as dual-chamber devices [17,18,19,20]. The limitations of these studies are small population numbers and, also, a difference in device manufacturers leading to differences in tachycardia discrimination algorithms [13]. In summary, the current guidelines do not facilitate the implantation of an extra atrial lead only for the purpose of better TA discrimination [31]. Our results are in line with this recommendation, since the non-inferiority of VVI ICD systems in appropriate anti-tachycardia therapy compared with the other two groups was demonstrated and there was no difference in the rate of inappropriate therapy as well. It is important to note, however, that DX systems offer the possibility to change the discrimination type between morphology and dual-chamber algorithms in the case of inappropriate therapy [7].

### 4.4. Long-Term Clinical Outcomes in Different ICD Types

Our results revealed no significant difference among the three groups regarding appropriate and inappropriate ICD therapies and all-cause mortality. While there was no difference in heart failure related hospitalization, we detected a significantly higher risk of arrhythmic hospitalization in the VDD ICD group compared with VVI ICDs. In this analysis, we also included hospital admission aiming at rhythm control strategies of atrial fibrillation (i.e., ECV, catheter ablation of atrial fibrillation/flutter) as an arrhythmic cause of hospitalization. As VDD ICDs were more effective in atrial arrhythmia detection, referrals to ECV/catheter ablation were consequently more frequent in this group, leading to higher hospitalization numbers and representing a better arrhythmia management (Table S14).

### 4.5. Limitations

Our data collection was retrospective; hence, all potential limitations of such a design apply to this analysis. To begin with, previously detected atrial fibrillation/atrial flutter might interfere the interpretation of EGMs. There were some differences in the baseline characteristics of the three groups because of the slight discrepancy in the composition of the DDD group. DDD ICD group involved proportionally higher number of patients with inherited cardiac diseases (e.g., hypertrophic cardiomyopathy, primer arrhythmic syndromes) compared with the other two groups and had a higher rate of conduction disturbances as well (Table 1, Tables S1 and S18). Accordingly, the higher number of patients with preserved/mildly reduced EF might also have an influence on the incidence of atrial arrhythmias in the DDD group. In addition, anatomical and operator-dependent factors, like atrial dimensions or the position of the floating dipole, might have influenced atrial sensing parameters, although these parameters were not collected. Furthermore, the difference in the rate of remote monitoring also forms a kind of bias, since it was most frequently applied in the VDD group, making early TA detection more efficient. While the increased use of remote monitoring may contribute to earlier atrial arrhythmia detection in VDD ICD patients, it might not impact the rate of arrhythmic hospitalizations, considering studies in the literature indicating that remote monitoring does not increase hospitalization rates; in fact, it may even reduce them in cases of worsening heart failure [32,33,34]. In Hungary, there is a heterogenous reimbursement for the different implantable ICD devices. For VDD ICDs, the cost of the remote monitor is included in the price of the device. Patients were implanted with devices manufactured by five different companies; therefore, we cannot exclude selection bias. Finally, new-generation VDD and DDD devices can be programmed to use single or dual-chamber discrimination to differentiate between atrial and ventricular TA. This information was not collected and, therefore, not included in the current analysis.

## 5. Conclusions

This single-center, retrospective study compared the TA detection capabilities of VDD ICD systems with those of conventional single- and dual-lead ICDs. Our findings demonstrate the superiority of VDD devices in atrial arrhythmia detection compared with VVI ICD systems and show non-inferiority compared to DDD devices. On the contrary, there were no difference in TA discrimination efficacy among the three groups. Based on the results of the superior atrial arrhythmia detection, the main indication of DX devices is to support optimal patient management and therapy for atrial arrhythmias rather than to improve TA discrimination. In patients who do not require atrial pacing, strong consideration should be given to use a VDD system in view of the valuable enhancement of atrial high-rate detection and the decrease in potential complications compared to DDD pacing by limiting the system to a single lead.

## Figures and Tables

**Table 1:** Baseline clinical characteristics.

	VVI (N = 93)	VDD (N = 94)	DDD (N = 69)	*p*-Value
Age (mean ± SD) ^1^	64 ± 12	63 ± 12	64 ± 14	0.307
Male (n, %)	65 (70%)	76 (81%)	50 (73%)	0.203
Primary prophylaxis (n, %)	19 (20%)	29 (31%)	24 (35%)	0.101
Ischemic etiology (n, %)	57 (61%)	58 (62%)	40 (59%)	0.926
Previously diagnosed atrial fibrillation (n, %)	27 (29%)	35 (37%)	25 (37%)	0.430
Hypertension (n, %)	86 (93%)	91 (97%)	60 (88%)	0.107
Dyslipidemia (n, %)	79 (85%)	85 (90%)	48 (71%)	**0.003**
Diabetes mellitus (n, %)	29 (31%)	33 (35%)	22 (33%)	0.850
Stroke/TIA (n, %)	11 (12%)	8 (9%)	9 (14%)	0.571
Bradypacing indication (n, %)	1 (1%)	3 (3%)	36 (55%)	**<0.001**
No bradypacing indication	92 (99%)	91 (97%)	30 (45,5%)	
Sick sinus syndrome	0	0	3 (4,5%)	
Second-degree AV block	1 (1%)	0	11 (16,7%)	
Third-degree AV block	0	2 (2%)	21 (31,8%)	
Atrial fibrillation with bradycardia	0	1 (1%)	1 (1,5%)	
Manufacturer (n, %) Biotronik Boston Scientific Medtronic SJM/Abott Sorin	2 (2%) 10 (11%) 45 (48%) 36 (39%) 0	92 (98%) 0 0 1 (1%) 1 (1%)	20 (29%) 9 (13%) 37 (54%) 3 (4%) 0	**<0.001**
NYHA (n, %) No NYHA I NYHA II NYHA III NYHA IV	30 (34%) 22 (25%) 25 (28%) 12 (14%) 0	34 (40%) 20 (24%) 18 (21%) 12 (14%) 1 (1%)	21 (33%) 17 (27%) 11 (18%) 13 (21%) 1 (2%)	0.707
LVEF (mean ± SD) ^2^	38.5 ± 13.5	37.5 ± 13.9	47.5 ± 16.3	**<0.001**
QRS width (mean ± SD) ^3^	116.9 ± 21.2	119 ± 21.6	136.9 ± 29.2	**0.001**
Heart rate (mean ± SD) ^4^	70.7 ± 15.4	73.1 ± 14.8	66.9 ± 18.6	**0.038**
Creatinine (mean ± SD) ^5^	91.1 ± 31.6	99.6 ± 52.9	94.9 ± 27.9	0.598
Hemoglobin (mean ± SD) ^6^	130.7 ± 20.6	136 ± 16.8	126.2 ± 20.7	**0.019**
Remote monitoring (n, %)	6 (7%)	48 (51%)	13 (19%)	**<0.001**

^1^ Available for 256 patients. Non-normal distribution, independent samples Kruskal–Wallis test. ^2^ Available for 249 patients. Non-normal distribution, independent samples Kruskal–Wallis test. ^3^ Available for 175 patients. Non-normal distribution, independent samples Kruskal–Wallis test. ^4^ Available for 229 patients. Non-normal distribution, independent samples Kruskal–Wallis test. ^5^ Available for 198 patients. Non-normal distribution, independent samples Kruskal–Wallis test. ^6^ Available for 196 patients. Non-normal distribution, independent samples Kruskal–Wallis test. VVI: conventional single-chamber ICD; VDD: single-lead ICD with a floating atrial dipole; DDD: conventional dual-chamber ICD; TIA: transient ischemic attack; SJM: Saint Jude Medical; NYHA: New York Heart Association; SD: standard deviation; LVEF: left ventricular ejection fraction.

**Table 2:** Baseline medical therapy.

	VVI (N = 93)	VDD (N = 94)	DDD (N = 69)	*p*-Value
Antiplatelet therapy (n, %)	62 (67%)	55 (59%)	41 (64%)	0.502
Anticoagulation (n, %)	33 (36%)	46 (49%)	26 (40%)	0.167
Beta-blockers (n, %)	90 (97%)	90 (96%)	53 (83%)	**0.001**
ACEI/ARB/ARNI (n, %)	81 (87%)	84 (89%)	50 (78%)	0.125
Diuretics (n, %)	50 (54%)	49 (52%)	28 (44%)	0.436
Calcium channel blockers (n, %)	17 (18%)	20 (21%)	19 (30%)	0.230
Mineralocorticoid receptor antagonists (n, %)	48 (52%)	52 (55%)	17 (27%)	**0.001**
Statins (n, %)	67 (72%)	66 (70%)	44 (69%)	0.903
Amiodarone (n, %)	17 (18%)	21 (22%)	10 (16%)	0.555
Digitalis glycosides (n, %)	13 (14%)	3 (3%)	3 (5%)	**0.012**
SGLT2 inhibitors (n, %)	2 (2%)	5 (5%)	3 (5%)	0.512

ACEI: angiotensin-converting-enzyme inhibitor; ARB: angiotensin II receptor blocker; ARNI: angiotensin receptor-neprilysin inhibitor; SGLT2: sodium glucose cotransporter 2.

**Table 3:** Long-term clinical outcomes.

	VVI vs. VDD	VDD vs. DDD
Time to first device detected atrial arrhythmia ^1^	HR (unadjusted) VVI vs. VDD 6.506; 95% CI 2.176–19.446; ***p* = 0.001** HR (adjusted) VVI vs. VDD 7.087; 95% CI 2.371–21.183; ***p* < 0.001**	HR (unadjusted) VDD vs. DDD 2.011; 95% CI 1.110–3.642; ***p* = 0.021** HR (adjusted) VDD vs. DDD 1.781; 95% CI 0.737–4.301; *p* = 0.200
Time to first appropriate therapy	HR (unadjusted) VVI vs. VDD 0.874; 95% CI 0.574–1.332; *p* = 0.523 HR (adjusted) VVI vs. VDD 0.983; 95% CI 0.641–1.508; *p* = 0.937	HR (unadjusted) VDD vs. DDD 0.611; 95% CI 0.359–1.040; *p* = 0.069 HR (adjusted) VDD vs. DDD 0.651; 95% CI 0.371–1.142; *p* = 0.135
Time to first inappropriate therapy	HR (unadjusted) VVI vs. VDD 0.782; 95% CI 0.338–1.811; *p* = 0.566 HR (adjusted) VVI vs. VDD 0.742; 95% CI 0.313–1.757; *p* = 0.497	HR (unadjusted) VDD vs. DDD 0.710; 95% CI 0.249–2.024; *p* = 0.522 HR (adjusted) VDD vs. DDD 0.618; 95% CI 0.203–1.878; *p* = 0.396
Time to first hospitalization due to arrhythmic cause	HR (unadjusted) VVI vs. VDD 1.463; 95% CI 0.899–2.379; *p* = 0.125 HR (adjusted) VVI vs. VDD 1.706; 95% CI 1.043–2.792; ***p* = 0.033**	HR (unadjusted) VDD vs. DDD 0.638; 95% CI 0.366–1.113; *p* = 0.114 HR (adjusted) VDD vs. DDD 0.700; 95% CI 0.365–1.341; *p* = 0.282
Time to first heart failure hospitalization	HR (unadjusted) VVI vs. VDD 0.949; 95% CI 0.449–2.006; *p* = 0.891 HR (adjusted) VVI vs. VDD 1.628; 95% CI 0.619–4.279; *p* = 0.323	HR (unadjusted) VDD vs. DDD 0.586; 95% CI 0.219–1.570; *p* = 0.287 HR (adjusted) VDD vs. DDD 0.949; 95% CI 0.301–2.991; *p* = 0.928
All-cause mortality	HR (unadjusted) VVI vs. VDD 0.914; 95% CI 0.543–1.537; *p* = 0.734	HR (unadjusted) VDD vs. DDD 0.812; 95% CI 0.442–1.490; *p* = 0.501
	HR (unadjusted) all groups 0.906; 95% CI 0.696–1.179; *p* = 0.463 HR (adjusted) all groups 0.960; 95% CI 0.711–1.295; *p* = 0.787	

^1^ In the time-to-event analysis of device detected atrial TA, patients with permanent atrial fibrillation were excluded. HR: hazard ratio; CI: confidence interval.

**Table 4:** Sensing/pacing parameters, and complication rates in the 3 ICD groups.

	VVI (N = 93)	VDD (N = 94)	DDD (N = 69)	*p*-Value
Atrial sensing at 6 months (mean ± SD) ^1^	N/A	5.32 ± 3.72	3.14 ± 2.09	**<0.001**
Atrial sensing at the end of the follow-up (mean ± SD) ^2^	N/A	4.15 ± 3.19	2.67 ± 1.81	**0.009**
Ventricular sensing at 6 months (mean ± SD) ^3^	12.76 ± 4.83	13.98 ± 6.00	13.17 ± 5.71	0.313
AP (mean ± SD) ^4^	N/A	N/A	22.58 ± 33.67	-
VP (mean ± SD) ^5^	2.16 ± 6.95	2.83 ± 14.40	33.61 ± 41.91	**<0.001**
Complications (n, %)	7 (8%)	12 (13%)	14 (20%)	0.056

^1^ Available for 127 patients. Non-normal distribution, independent samples Mann–Whitney U test. ^2^ Available for 144 patients. Non-normal distribution, independent samples Mann–Whitney U test. ^3^ Available for 239 patients. Non-normal distribution, independent samples Kruskal–Wallis test. ^4^ Available for 55 patients. ^5^ Available for 246 patients. Non-normal distribution independent samples Kruskal–Wallis test. N/A: not applicable; AP: atrial pace; VP: ventricular pace.

## Data Availability

All data used in this study are available by reasonable request from the corresponding author.

## Supplementary Materials

The following supporting information can be downloaded at: https://www.mdpi.com/article/10.3390/jcdd11120386/s1, Table S1: Subgroup comparisons of baseline characteristics; Table S2: Subgroup comparisons of baseline medical therapy; Table S3: Multivariate analysis of time to first device detected atrial arrhythmia—VVI vs. VDD; Table S4: Multivariate analysis of time to first device detected atrial arrhythmia—VDD vs. DDD; Table S5: Detailed distribution of device detected atrial arrhythmias; Table S6: Subgroup analysis of complication rates; Table S7: Distribution of complications between all groups; Table S8: Multivariate analysis of time to first appropriate therapy—VVI vs. VDD; Table S9: Multivariate analysis of time to first appropriate therapy -VDD vs. DDD; Table S10: Multivariate analysis of time to first inappropriate therapy—VVI vs. VDD; Table S11: Multivariate analysis of time to first inappropriate therapy—VDD vs. DDD; Table S12: Multivariate analysis of time to first hospitalization due to arrhythmic cause—VVI vs. VDD; Table S13: Multivariate analysis of time to first hospitalization due to arrhythmic cause—VDD vs. DDD; Table S14: Arrhythmia-related hospitalization events in the different groups; Table S15: Multivariate analysis of time to first hospitalization due to heart failure—VVI vs. VDD; Table S16: Multivariate analysis of time to first hospitalization due to heart failure—VDD vs. DDD; Table S17: Multivariate analysis of all-cause mortality—all groups; Table S18: Distribution of structural heart diseases between all groups; Figure S1: Ventricular sensing at 6th month; Figure S2: A: Time to first appropriate therapy—VVI vs. VDD; B: Time to first appropriate therapy—VDD vs. DDD; C: Time to first inappropriate therapy—VVI vs. VDD; D: Time to first inappropriate therapy—VDD vs. DDD; Figure S3: A: Time to first hospitalization due to arrhythmic cause—VVI vs. VDD; B: Time to first hospitalization due to arrhythmic cause—VDD vs. DDD; Figure S4: A: Time to first heart failure (HF) hospitalization—VVI vs. VDD; B: Time to first HF hospitalization—VDD vs. DDD.

## References

[1] Zeppenfeld K., Tfelt-Hansen J., de Riva M., Winkel B.G., Behr E.R., Blom N.A., Charron P., Corrado D., Dagres N., de Chillou C. (2022). 2022 ESC Guidelines for the management of patients with ventricular arrhythmias and the prevention of sudden cardiac death. Eur. Heart J..

[2] Stroobandt R.X., Barold S.S., Sinnaeve A.F. (2011). Implantable Cardioverter-Defibrillators Step by Step: An Illustrated Guide.

[3] Tzeis S., Gerstenfeld E.P., Kalman J., Saad E.B., Sepehri Shamloo A., Andrade J.G., Barbhaiya C.R., Baykaner T., Boveda S., Calkins H. (2024). 2024 European Heart Rhythm Association/Heart Rhythm Society/Asia Pacific Heart Rhythm Society/Latin American Heart Rhythm Society expert consensus statement on catheter and surgical ablation of atrial fibrillation. EP Eur..

[4] Hindricks G., Theuns D.A., Bar-Lev D., Anguera I., Ayala Paredes F.A., Arnold M., Geller J.C., Merkely B., Dyrda K.M., Perings C. (2023). Ability to remotely monitor atrial high-rate episodes using a single-chamber implantable cardioverter-defibrillator with a floating atrial sensing dipole. Europace.

[5] Benz A.P., McIntyre W.F. (2024). Oral factor Xa inhibitors for stroke prevention in patients with device-detected atrial fibrillation—Recent evidence from the NOAH-AFNET 6 and ARTESiA trials. Cardiol. Hung..

[6] Thomas G., Choi D.Y., Doppalapudi H., Richards M., Iwai S., Daoud E.G., Houmsse M., Kanagasundram A.N., Mainigi S.K., Lubitz S.A. (2019). Subclinical atrial fibrillation detection with a floating atrial sensing dipole in single lead implantable cardioverter-defibrillator systems: Results of the SENSE trial. J. Cardiovasc. Electrophysiol..

[7] Vamos M., Nemeth M., Balazs T., Zima E., Duray G.Z. (2022). Rationale and feasibility of the atrioventricular single-lead ICD systems with a floating atrial dipole (DX) in clinical practice. Trends Cardiovasc. Med..

[8] Kurt M., Jathanna N., Babady M., Schmidt J., Müller P., Gerguri S., Clasen L., Bejinariu A., Kelm M., Fürnkranz A. (2018). Avoiding inappropriate therapy of single-lead implantable cardioverter-defibrillator by using atrial-sensing electrodes. J. Cardiovasc. Electrophysiol..

[9] Pung X., Hong D.Z., Ho T.Y., Shen X., Tan P.T., Yeo C., Tan V.H. (2022). The utilization of atrial sensing dipole in single lead implantable cardioverter defibrillator for detection of new-onset atrial high-rate episodes or subclinical atrial fibrillation: A systematic review and meta-analysis. J. Arrhythm..

[10] Worden N.E., Alqasrawi M., Krothapalli S.M., Mazur A. (2016). “Two for the Price of One”: A Single-Lead Implantable Cardioverter-Defibrillator System with a Floating Atrial Dipole. J. Atr. Fibrillation.

[11] Jacheć W., Nowosielecka D., Ziaja B., Polewczyk A., Kutarski A. (2023). LECOM (Lead Extraction COMplexity): A New Scoring System for Predicting a Difficult Procedure. J. Clin. Med..

[12] Biffi M., Iori M., De Maria E., Bolognesi M.G., Placci A., Calvi V., Allocca G., Ammendola E., Carinci V., Boggian G. (2020). The role of atrial sensing for new-onset atrial arrhythmias diagnosis and management in single-chamber implantable cardioverter-defibrillator recipients: Results from the THINGS registry. J. Cardiovasc. Electrophysiol..

[13] O’Connor M., Kolb C., Klein N., Rauwolf T., Kuster S., Kääb S., Tilz R.R., Bänsch D., Ince H., Belke R. (2023). REACT DX registry: Real world REACTion to atrial high rate episodes detected in implantable cardioverter-defibrillator recipients with a DX lead. Technol. Health Care.

[14] Brüggemann T., Dahlke D., Chebbo A., Neumann I. (2016). Tachycardia detection in modern implantable cardioverter-defibrillators. Herzschrittmacherther. Elektrophysiol..

[15] Briongos-Figuero S., Sánchez A., Pérez M.L., Martínez-Ferrer J.B., García E., Viñolas X., Arenal Á., Alzueta J., Basterra N., Rodríguez A. (2019). Single-brand dual-chamber discriminators to prevent inappropriate shocks in patients implanted with prophylactic implantable cardioverter defibrillators: A propensity-weighted comparison of single- and dual-chamber devices. J. Interv. Card. Electrophysiol..

[16] Kolb C., Sturmer M., Sick P., Reif S., Davy J.M., Molon G., Schwab J.O., Mantovani G., Dan D., Lennerz C. (2014). Reduced risk for inappropriate implantable cardioverter-defibrillator shocks with dual-chamber therapy compared with single-chamber therapy: Results of the randomized OPTION study. JACC Heart Fail..

[17] Gold M.R., Ahmad S., Browne K., Berg K.C., Thackeray L., Berger R.D. (2012). Prospective comparison of discrimination algorithms to prevent inappropriate ICD therapy: Primary results of the Rhythm ID Going Head to Head Trial. Heart Rhythm..

[18] Friedman P.A., Bradley D., Koestler C., Slusser J., Hodge D., Bailey K., Kusumoto F., Munger T.M., Militanu A., Glikson M. (2014). A prospective randomized trial of single- or dual-chamber implantable cardioverter-defibrillators to minimize inappropriate shock risk in primary sudden cardiac death prevention. Europace.

[19] Peterson P.N., Greenlee R.T., Go A.S., Magid D.J., Cassidy-Bushrow A., Garcia-Montilla R., Glenn K.A., Gurwitz J.H., Hammill S.C., Hayes J. (2017). Comparison of Inappropriate Shocks and Other Health Outcomes Between Single- and Dual-Chamber Implantable Cardioverter-Defibrillators for Primary Prevention of Sudden Cardiac Death: Results from the Cardiovascular Research Network Longitudinal Study of Implantable Cardioverter-Defibrillators. J. Am. Heart Assoc..

[20] Németh M., Zima E.I., Duray G.Z., Balázs T., Vámos M. (2021). Atrioventrikuláris, egyelektródás, lebegő pitvari dipólussal rendelkező defibrillátorok (DX ICD) a klinikai gyakorlatban [Clinical utility of the atrioventricular, single-lead defibrillator systems with a floating atrial dipole (DX ICD): An executive summary]. Cardiol. Hung..

[21] Hindricks G., Potpara T., Dagres N., Arbelo E., Bax J.J., Blomström-Lundqvist C., Boriani G., Castella M., Dan G.A., Dilaveris P.E. (2021). 2020 ESC Guidelines for the diagnosis and management of atrial fibrillation developed in collaboration with the European Association for Cardio-Thoracic Surgery (EACTS): The Task Force for the diagnosis and management of atrial fibrillation of the European Society of Cardiology (ESC) Developed with the special contribution of the European Heart Rhythm Association (EHRA) of the ESC. Eur. Heart J..

[22] Vamos M., Nemeth M., Kesoi B., Papp R., Polgar B., Ruppert M., Mikler C., Liptak A., Selley T., Balazs T. (2024). Key influences of VDD (DX) ICD selection: Results from a prospective, national survey. Pacing Clin. Electrophysiol..

[23] Shurrab M., Janmohamed A.K., Ayala-Paredes F.A., Sturmer M., Toal S.C., Sarrazin J.-F., Thorpe K.E., Sterns L.D., Healey J.S., Crystal E. (2024). A Prospective, Multicenter, Randomized Controlled Trial Comparing VDD-ICD to VVI-ICD in Detecting Sub-Clinical Atrial Fibrillation in Patients with ICDs: The Dx-AF trial. Heart Rhythm O2.

[24] Healey J.S., Connolly S.J., Gold M.R., Israel C.W., Van Gelder I.C., Capucci A., Lau C.P., Fain E., Yang S., Bailleul C. (2012). Subclinical atrial fibrillation and the risk of stroke. N. Engl. J. Med..

[25] Steinberg B.A., Piccini J.P. (2018). When Low-Risk Atrial Fibrillation Is Not So Low Risk: Beast of Burden. JAMA Cardiol..

[26] Kirchhof P., Toennis T., Goette A., Camm A.J., Diener H.C., Becher N., Bertaglia E., Blomstrom Lundqvist C., Borlich M., Brandes A. (2023). Anticoagulation with Edoxaban in Patients with Atrial High-Rate Episodes. N. Engl. J. Med..

[27] Lopes R.D., Alings M., Connolly S.J., Beresh H., Granger C.B., Mazuecos J.B., Boriani G., Nielsen J.C., Conen D., Hohnloser S.H. (2017). Rationale and design of the Apixaban for the Reduction of Thrombo-Embolism in Patients with Device-Detected Sub-Clinical Atrial Fibrillation (ARTESiA) trial. Am. Heart J..

[28] McIntyre W.F., Benz A.P., Becher N., Healey J.S., Granger C.B., Rivard L., Camm A.J., Goette A., Zapf A., Alings M. (2024). Direct Oral Anticoagulants for Stroke Prevention in Patients with Device-Detected Atrial Fibrillation: A Study-Level Meta-Analysis of the NOAH-AFNET 6 and ARTESiA Trials. Circulation.

[29] Mukherjee R.K., Sohal M., Shanmugam N., Pearse S., Jouhra F. (2021). Successful Identification of and Discrimination Between Atrial and Ventricular Arrhythmia with the Aid of Pacing and Defibrillator Devices. Arrhythm. Electrophysiol. Rev..

[30] Cheng A., Auricchio A., Schloss E.J., Kurita T., Sterns L.D., Gerritse B., Brown M.L., Fagan D.H., Lexcen D.R., Ellenbogen K.A. (2019). SVT discrimination algorithms significantly reduce the rate of inappropriate therapy in the setting of modern-day delayed high-rate detection programming. J. Cardiovasc. Electrophysiol..

[31] Wilkoff B.L., Fauchier L., Stiles M.K., Morillo C.A., Al-Khatib S.M., Almendral J., Aguinaga L., Berger R.D., Cuesta A., Daubert J.P. (2016). 2015 HRS/EHRA/APHRS/SOLAECE expert consensus statement on optimal implantable cardioverter-defibrillator programming and testing. EP Eur..

[32] Reinhardt A., Ventura R. (2023). Remote Monitoring of Cardiac Implantable Electronic Devices: What is the Evidence?. Curr. Heart Fail. Rep..

[33] Parthiban N., Esterman A., Mahajan R., Twomey D.J., Pathak R.K., Lau D.H., Roberts-Thomson K.C., Young G.D., Sanders P., Ganesan A.N. (2015). Remote Monitoring of Implantable Cardioverter-Defibrillators: A Systematic Review and Meta-Analysis of Clinical Outcomes. J. Am. Coll. Cardiol..

[34] Hindricks G., Varma N., Kacet S., Lewalter T., Søgaard P., Guedon-Moreau L., Proff J., Gerds T.A., Anker S.D., Torp-Pedersen C. (2017). Daily remote monitoring of implantable cardioverter-defibrillators: Insights from the pooled patient-level data from three randomized controlled trials (IN-TIME, ECOST, TRUST). Eur. Heart J..

