# Supplementary material for: Arrhythmia Detection in Atrioventricular, Single-Lead, Floating Atrial Dipole ICD Systems Compared with Conventional Single- and Dual-Chamber Defibrillators
Source: J Cardiovasc Dev Dis. 2024 Dec 1;11(12):386. doi: 10.3390/jcdd11120386 (PMC11677019; doi:10.3390/jcdd11120386)
Supplement: Supplementary file 1 [file jcdd-11-00386-s001.zip › jcdd-3195363-supplementary.pdf]

Journal of
Cardiovascular Development and Disease

MDPI

Arrhythmia detection in atrioventricular, single-lead, floating atrial dipole ICD systems compared to conventional single- and dual-chamber defibrillators

Supplementary material

Table S1. Subgroup comparisons of baseline characteristics

	**VVI (N=93)**	**VDD (N=94)**	**p-value**
Dyslipidaemia (n, %)	79 (85%)	85 (90%)	0.254
LVEF (mean±SD)[1]	38.5±13.5	37.5±13.9	0.642
QRS width (mean±SD)[2]	116.9±21.2	119±21.6	0.909
Heart rate (mean±SD)[3]	70.7±15.4	73.1±14.8	0.249
Bradypacing indication (n, %)	1 (1%)	3 (3%)	0.317
Hemoglobin (mean±SD)[4]	130.7±20.6	136±16.8	0.062
Remote monitoring (n, %)	6 (7%)	48 (51%)	**<0.001**
	**VVI (N=93)**	**DDD (N=69)**	**p-value**
Dyslipidaemia (n, %)	79 (85%)	48 (71%)	**0.027**
LVEF (mean±SD)[1]	38.5±13.5	47.5±16.3	**<0.001**
QRS width (mean±SD)[2]	116.9±21.2	136.9±29.2	**<0.001**
Heart rate (mean±SD)[3]	70.7±15.4	66.9±18.6	0.117
Bradypacing indication (n, %)	1 (1%)	36 (55%)	**<0.001**
Hemoglobin (mean±SD)[4]	130.7±20.6	126.2±20.7	0.237
Remote monitoring (n, %)	6 (7%)	13 (19%)	**0.014**
	**VDD (N=94)**	**DDD (N=69)**	**p-value**
Dyslipidaemia (n, %)	85 (90%)	48 (71%)	**0.001**
LVEF (mean±SD)[1]	37.5±13.9	47.5±16.3	**0.001**
QRS width (mean±SD)[2]	119±21.6	136.9±29.2	**0.001**
Heart rate (mean±SD)[3]	73.1±14.8	66.9±18.6	**0.011**
Bradypacing indication (n, %)	3 (3%)	36 (55%)	**<0.001**
Hemoglobin (mean±SD)[4]	136±16.8	126.2±20.7	**0.008**
Remote monitoring (n, %)	48 (51%)	13 (19%)	**<0.001**

[1] Non-normal distribution, independent samples Mann-Whitney U test.
[2] Non-normal distribution, independent samples Mann-Whitney U test.
[3] Non-normal distribution, independent samples Mann-Whitney U test.
[4] VVI vs. VDD: Non-normal distribution, independent samples Mann-Whitney U test, VVI vs. DDD: Normal distribution, independent samples t-test, VDD vs. DDD: Non-normal distribution, independent samples Mann-Whitney U test.
VVI: conventional single-chamber ICD; VDD: single-lead ICD device with a floating atrial dipole; DDD: conventional dual-chamber ICD; SD: standard deviation; LVEF: left ventricular ejection fraction

Table S2. Subgroup comparisons of baseline medical therapy

	**VVI (N=93)**	**VDD (N=94)**	**p-value**
Beta-blockers (n, %)	90 (97%)	90 (96%)	0.711
Mineralocorticoid receptor antagonists (n, %)	48 (52%)	52 (55%)	0.611
Digitalis glycosides (n, %)	13 (14%)	3 (3%)	**0.008**
	**VVI (N=93)**	**DDD (N=69)**	**p-value**
Beta-blockers (n, %)	90 (97%)	53 (83%)	**0.003**
Mineralocorticoid receptor antagonists (n, %)	48 (52%)	17 (27%)	**0.002**
Digitalis glycosides (n, %)	13 (14%)	3 (5%)	0.059
	**VDD (N=94)**	**DDD (N=69)**	**p-value**
Beta-blockers (n, %)	90 (96%)	53 (83%)	**0.006**
Mineralocorticoid receptor antagonists (n, %)	52 (55%)	17 (27%)	**<0.001**
Digitalis glycosides (n, %)	3 (3%)	3 (5%)	0.629

Table S3. Multivariate analysis of time to first device detected atrial arrhythmia **- VVI vs. VDD**

**Risk factor**	**unadjusted HR (95% CI)**	**p-value**	**adjusted HR (95% CI)**	**p-value**
ICD type	6.506 (2.176-19.446)	**0.001**	7.087 (2.371-21.183)	**<0.001**
Age	1.004 (0.969-1.040)	0.837		
Male	0.540 (0.226-1.291)	0.166		
Primary prophylaxis	6.989 (0.940-51.970)	**0.058**	9.746 (1.301-73.014)	**0.027**
Ischemic etiology	1.561 (0.609-4.002)	0.354		
Previously diagnosed atrial fibrillation	1.916 (0.743-4.942)	0.179		
Hypertension	2.280 (0.303-17.126)	0.423		
Dyslipidaemia	4.127 (0.554-30.776)	0.167		
Diabetes mellitus	1.456 (0.609-3.478)	0.398		
Stroke/TIA	0.043 (0.000-28.860)	0.343		
Bradypacing indication	7.895 (0.994-62.697)	**0.051**	18.471 (1.973-172.887)	**0.011**
LVEF	0.981 (0.947-1.015)	0.270		
QRS width	1.009 (0.986-1.033)	0.442		
Heart rate	1.033 (1.001-1.066)	**0.042**	1.038 (1.004-1.074)	**0.028**
Creatinine	1.003 (0.996-1.009)	0.473		
Hemoglobin	1.003 (0.978-1.029)	0.809		
Remote monitoring	2.628 (1.133-6.093)	**0.024**	0.907 (0.347-2.368)	0.841
Antiplatelet therapy	1.013 (0.412-2.489)	0.977		
Anticoagulation	1.609 (0.686-3.770)	0.274		
Beta-blockers	0.682 (0.092-5.075)	0.709		
ACEI/ARB/ARNI	0.550 (0.186-1.627)	0.280		
Diuretics	1.564 (0.668-3.661)	0.303		
Calcium channel blockers	0.884 (0.299-2.616)	0.824		

Mineralocorticoid receptor antagonists	1.020 (0.441-2.358)	0.963		
Statins	1.057 (0.412-2.709)	0.909		
Amiodarone	1.285 (0.474-3.483)	0.623		
Digitalis glycosides	0.045 (0.000-116.944)	0.440		

HR: hazard ratio; CI: confidence interval; ICD: implantable cardioverter defibrillator; TIA: transient ischemic attack; ACEI: angiotensin-converting-enzyme inhibitor; ARB: angiotensin II receptor blocker; ARNI: angiotensin receptor-neprilysin inhibitor

Table S4. Multivariate analysis of time to first device detected atrial arrhythmia **- VDD vs. DDD**

**Risk factor**	**unadjusted HR (95% CI)**	**p-value**	**adjusted HR (95% CI)**	**p-value**
ICD type	2.011 (1.110-3.642)	**0.021**	1.781 (0.737-4.301)	0.200
Age	1.038 (1.012-1.066)	**0.004**	0.992 (0.959-1.026)	0.645
Male	0.731 (0.384-1.390)	0.339		
Primary prophylaxis	1.951 (0.941-4.044)	**0.072**	1.001 (0.379-2.643)	0.998
Ischemic etiology	1.522 (0.816-2.838)	0.187		
Previously diagnosed atrial fibrillation	3.822 (2.085-7.005)	**<0.001**	4.300 (1.687-10.959)	**0.002**
Hypertension	3.185 (0.765-13.260)	0.111		
Dyslipidaemia	1.334 (0.617-2.881)	0.464		
Diabetes mellitus	1.344 (0.735-2.458)	0.337		
Stroke/TIA	0.451 (0.109-1.867)	0.272		
Bradypacing indication	2.195 (1.207-3.992)	**0.010**	0.749 (0.216-2.601)	0.649
LVEF	0.999 (0.981-1.018)	0.950		
QRS width	1.013 (0.999-1.026)	**0.065**	0.998 (0.979-1.017)	0.833
Heart rate	1.019 (0.998-1.041)	**0.075**	1.008 (0.974-1.043)	0.668
Creatinine	1.002 (0.997-1.007)	0.470		
Hemoglobin	0.995 (0.977-1.013)	0.582		
Remote monitoring	0.388 (0.200-0.751)	**0.005**	0.323 (0.123-0.852)	**0.022**
Antiplatelet therapy	1.139 (0.609-2.131)	0.684		
Anticoagulation	2.723 (1.506-4.924)	**0.001**	0.746 (0.295-1.883)	0.535
Beta-blockers	1.685 (0.520-5.456)	0.384		
ACEI/ARB/ARNI	1.043 (0.482-2.255)	0.915		
Diuretics	1.731 (0.953-3.147)	**0.072**	4.105 (1.610-10.462)	**0.003**
Calcium channel blockers	1.043 (0.527-2.066)	0.903		
Mineralocorticoid receptor antagonists	0.752 (0.403-1.405)	0.371		
Statins	1.245 (0.638-2.431)	0.520		
Amiodarone	1.901 (0.930-3.886)	**0.078**	1.178 (0.403-3.447)	0.765
Digitalis glycosides	3.043 (0.731-12.672)	0.126		

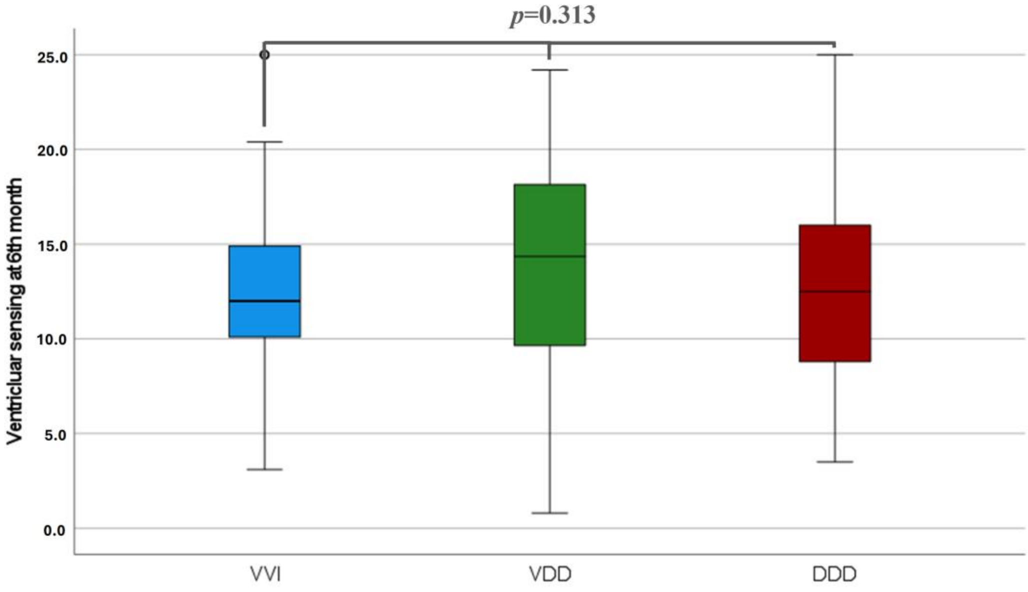

Figure S1 Ventricular sensing in VVI vs. VDD vs. DDD ICDs at 6th month

Table S5. Detailed distribution of device detected atrial arrhythmias

	VVI (N=93)	VDD (N=94)	DDD (N=69)	p-value
Total number of first device detected atrial arrhythmias	4	18	28	
Paroxysmal atrial fibrillation (n, %)	4 (100%)	15 (83%)	23 (82%)	
Persistent atrial fibrillation (n, %)	0 (0%)	2 (11%)	1 (4%)	0.609
Regular atrial arrhythmias (n, %)	0 (0%)	1 (6%)	4 (14%)	

Table S6. Subgroup analysis of complication rates

	VVI (N=93)	VDD (N=94)	p-value
Complications (n, %)	7 (8%)	12 (13%)	0.236
	**VVI (N=93)**	**DDD (N=69)**	**p-value**
Complications (n, %)	7 (8%)	14 (20%)	**0.017**
	**VDD (N=94)**	**DDD (N=69)**	**p-value**
Complications (n, %)	12 (13%)	14 (20%)	0.195

Table S7. Distribution of complications between all groups

	**VVI (N=93)**	**VDD (N=94)**	**DDD (N=69)**
Superficial infection (n,%)	2 (2%)	5 (5%)	3 (4%)
Pocket infection (n,%)	1 (1%)	0 (0%)	2 (3%)
Systemic infection (n,%)	2 (2%)	0 (0%)	1 (1%)
Hematoma (n,%)	1 (1%)	2 (2%)	2 (3%)
Deep venous thrombosis (n,%)	0 (0%)	0 (0%)	1 (1%)
Lead-related complication (n,%)	1 (1%)	5 (5%)	5 (7%)

Table S8. Multivariate analysis of time to first appropriate therapy **- VVI vs. VDD**

**Risk factor**	**unadjusted HR (95% CI)**	**p-value**	**adjusted HR (95% CI)**	**p-value**
ICD type	0.874 (0.574-1.332)	**0.532**	0.983 (0.641-1.508)	0.937
Age	1.023 (1.004-1.043)	**0.019**	1.019 (0.999-1.039)	0.057
Male	1.127 (0.691-1.838)	0.631		
Primary prophylaxis	2.760 (1.533-4.972)	**0.001**	2.204 (1.192-4.074)	**0.012**
Ischemic etiology	0.893 (0.586-1.359)	0.597		
Previously diagnosed atrial fibrillation	0.920 (0.577-1.469)	0.728		
Hypertension	1.050 (0.483-2.281)	0.902		
Dyslipidaemia	1.275 (0.678-2.398)	0.450		
Diabetes mellitus	0.812 (0.512-1.287)	0.375		
Stroke/TIA	0.942 (0.455-1.950)	0.873		
Bradypacing indication	0.634 (0.088-4.562)	0.651		
LVEF	1.000 (0.985-1.014)	0.949		
QRS width	1.003 (0.991-1.014)	0.655		
Heart rate	0.993 (0.978-1.009)	0.376		
Creatinine	1.003 (0.998-1.008)	0.194		
Hemoglobin	1.000 (0.987-1.014)	0.950		
Remote monitoring	0.895 (0.565-1.419)	0.637		
Antiplatelet therapy	0.882 (0.577-1.349)	0.563		
Anticoagulation	0.827 (0.540-1.266)	0.381		
Beta-blockers	0.255 (0.103-0.636)	**0.003**	0.256 (0.101-0.648)	**0.004**
ACEI/ARB/ARNI	1.140 (0.572-2.271)	0.710		
Diuretics	1.172 (0.775-1.772)	0.452		
Calcium channel blockers	1.736 (1.088-2.770)	**0.021**	1.352 (0.837-2.184)	0.218
Mineralocorticoid receptor antagonists	0.975 (0.646-1.473)	0.906		
Statins	0.719 (0.464-1.114)	0.139		
Amiodarone	1.691 (1.051-2.721)	**0.030**	1.521 (0.926-2.498)	0.098
Digitalis glycosides	0.618 (0.270-1.416)	0.255		

Table S9. Multivariate analysis of time to first appropriate therapy **- VDD vs. DDD**

**Risk factor**	**unadjusted HR (95% CI)**	**p-value**	**adjusted HR (95% CI)**	**p-value**
ICD type	0.611 (0.359-1.040)	**0.069**	0.651 (0.371-1.142)	0.135
Age	1.012 (0.991-1.033)	0.240		
Male	1.088 (0.597-1.983)	0.784		
Primary prophylaxis	3.488 (1.714-7.097)	**<0.001**	3.341 (1.564-7.138)	**0.002**
Ischemic etiology	1.330 (0.782-2.262)	0.293		
Previously diagnosed atrial fibrillation	0.954 (0.548-1.663)	0.869		
Hypertension	1.353 (0.489-3.745)	0.560		
Dyslipidaemia	1.645 (0.779-3.473)	0.192		
Diabetes mellitus	0.785 (0.446-1.382)	0.401		
Stroke/TIA	0.641 (0.232-1.771)	0.391		
Bradypacing indication	0.523 (0.265-1.035)	**0.063**	0.721 (0.315-1.650)	0.439
LVEF	0.992 (0.976-1.008)	0.336		
QRS width	0.999 (0.987-1.011)	0.883		
Heart rate	0.993 (0.974-1.011)	0.438		
Creatinine	1.002 (0.996-1.008)	0.560		
Hemoglobin	1.002 (0.986-1.018)	0.822		
Remote monitoring	1.420 (0.850-2.371)	0.181		
Antiplatelet therapy	0.898 (0.529-1.526)	0.691		
Anticoagulation	1.269 (0.752-2.141)	0.371		
Beta-blockers	1.301 (0.470-3.603)	0.612		
ACEI/ARB/ARNI	0.971 (0.476-1.983)	0.936		
Diuretics	1.146 (0.681-1.927)	0.608		
Calcium channel blockers	1.483 (0.840-2.617)	0.174		
Mineralocorticoid receptor antagonists	0.962 (0.562-1.647)	0.888		
Statins	0.912 (0.521-1.594)	0.745		
Amiodarone	2.480 (1.374-4.479)	**0.003**	1.949 (1.073-3.540)	**0.028**
Digitalis glycosides	0.046 (0.000-11.224)	0.272		

Table S10. Multivariate analysis of time to first inappropriate therapy **- VVI vs. VDD**

Risk factor	unadjusted HR (95% CI)	p-value	adjusted HR (95% CI)	p-value
ICD type	0.782 (0.338-1.811)	**0.566**	0.742 (0.313-1.757)	0.497
Age	0.994 (0.960-1.029)	0.729		
Male	0.613 (0.262-1.434)	0.259		
Primary prophylaxis	0.938 (0.372-2.365)	0.892		
Ischemic etiology	0.527 (0.236-1.178)	0.119		
Previously diagnosed atrial fibrillation	1.111 (0.459-2.689)	0.816		
Hypertension	0.588 (0.173-1.996)	0.395		
Dyslipidaemia	0.332 (0.142-0.777)	**0.011**	0.533 (0.213-1.332)	0.178
Diabetes mellitus	0.592 (0.221-1.586)	0.297		
Stroke/TIA	0.043 (0.000-24.731)	0.332		
Bradypacing indication	0.048 (0.000-132902.528)	0.689		
LVEF	0.984 (0.952-1.016)	0.322		
QRS width	1.006 (0.982-1.031)	0.637		
Heart rate	1.007 (0.980-1.034)	0.628		
Creatinine	1.002 (0.996-1.009)	0.482		
Hemoglobin	1.011 (0.984-1.039)	0.430		
Remote monitoring	0.717 (0.284-1.808)	0.480		
Antiplatelet therapy	0.365 (0.161-0.824)	**0.015**	0.327 (0.142-0.753)	**0.009**
Anticoagulation	1.519 (0.677-3.410)	0.311		
Beta-blockers	0.240 (0.056-1.026)	**0.054**	0.167 (0.037-0.742)	**0.019**
ACEI/ARB/ARNI	0.406 (0.151-1.088)	**0.073**	0.667 (0.214-2.078)	0.485
Diuretics	1.419 (0.630-3.197)	0.398		
Calcium channel blockers	0.525 (0.156-1.761)	0.297		
Mineralocorticoid receptor antagonists	1.131 (0.506-2.530)	0.764		
Statins	0.464 (0.207-1.036)	**0.061**	1.158 (0.339-3.955)	0.815
Amiodarone	0.938 (0.321-2.747)	0.908		
Digitalis glycosides	1.602 (0.477-5.377)	0.446		

Table S11. Multivariate analysis of time to first inappropriate therapy **- VDD vs. DDD**

Risk factor	unadjusted HR (95% CI)	p-value	adjusted HR (95% CI)	p-value
ICD type	0.710 (0.249-2.024)	**0.522**	0.618 (0.203-1.878)	0.396
Age	0.986 (0.955-1.019)	0.399		
Male	1.396 (0.397-4.907)	0.603		
Primary prophylaxis	1.927 (0.548-6.774)	0.307		
Ischemic etiology	0.483 (0.179-1.306)	0.152		
Previously diagnosed atrial fibrillation	1.703 (0.599-4.839)	0.318		
Hypertension	1.753 (0.229-13.404)	0.589		
Dyslipidaemia	1.090 (0.309-3.847)	0.893		
Diabetes mellitus	0.624 (0.199-1.957)	0.418		
Stroke/TIA	0.042 (0.000-58.362)	0.390		
Bradypacing indication	0.383 (0.086-1.705)	0.208		
LVEF	0.988 (0.956-1.022)	0.491		
QRS width	1.000 (0.974-1.025)	0.971		
Heart rate	0.974 (0.936-1.014)	0.197		
Creatinine	1.002 (0.994-1.011)	0.558		
Hemoglobin	1.030 (0.988-1.074)	0.158		
Remote monitoring	0.783 (0.284-2.159)	0.636		
Antiplatelet therapy	0.484 (0.175-1.340)	0.162		
Anticoagulation	2.314 (0.796-6.723)	0.123		
Beta-blockers	0.751 (0.168-3.366)	0.708		
ACEI/ARB/ARNI	0.843 (0.237-3.001)	0.792		
Diuretics	1.754 (0.623-4.937)	0.287		
Calcium channel blockers	1.018 (0.323-3.212)	0.976		
Mineralocorticoid receptor antagonists	2.211 (0.761-6.424)	0.145		
Statins	0.606 (0.215-1.708)	0.344		
Amiodarone	1.457 (0.404-5.249)	0.565		
Digitalis glycosides	5.246 (1.169-23.545)	**0.030**	5.246 (1.169-23.545)	**0.030**

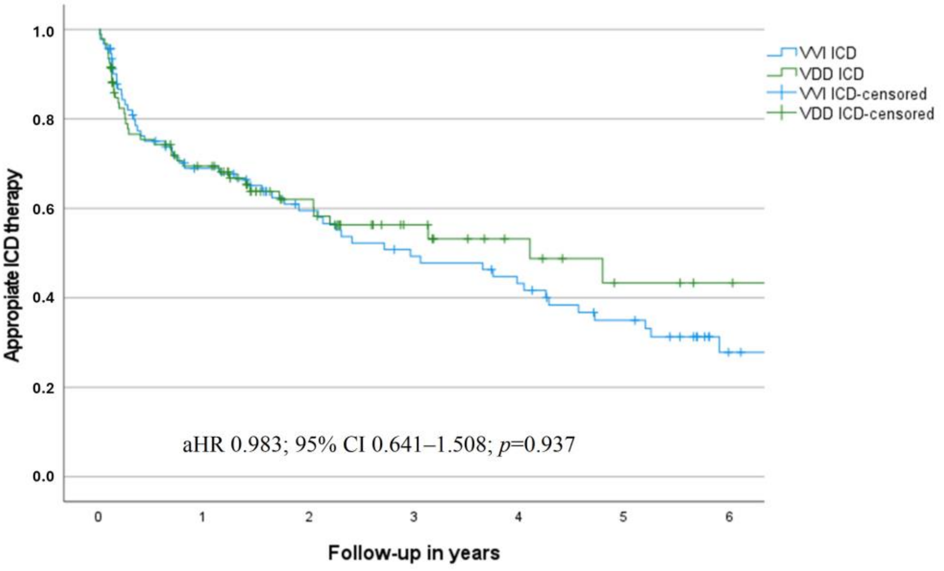

Patients at risk	1 year	2 years	3 years	4 years	5 years	6 years	7 years
**VVI**	93	55	41	33	28	20	7
**VDD**	94	56	33	19	12	7	5

Figure S2A Time to first appropriate therapy – VVI vs. VDD

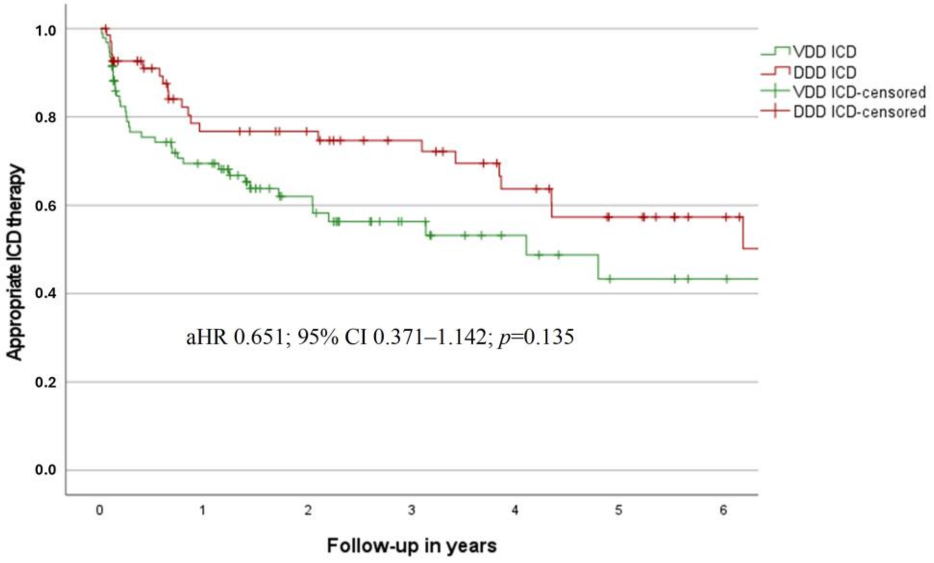

Patients at risk	1 year	2 years	3 years	4 years	5 years	6 years	7 years
**VDD**	94	56	33	19	12	7	5
**DDD**	69	42	37	30	22	16	10

Figure S2B Time to first appropriate therapy – VDD vs. DDD

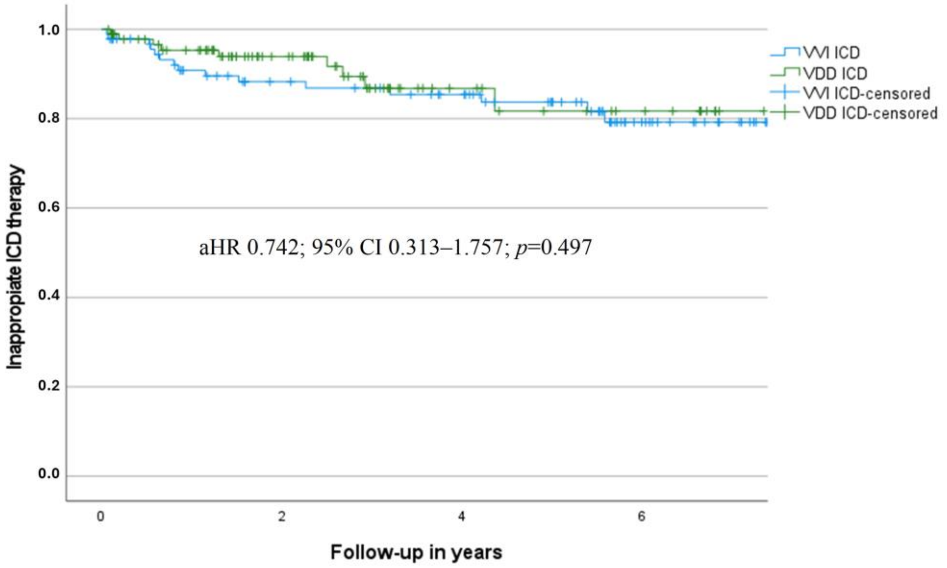

Patients at risk	1 year	2 years	3 years	4 years	5 years	6 years	7 years	8 years
**VVI**	93	73	64	61	55	44	23	13
**VDD**	94	75	52	30	19	14	11	1

Figure S2C Time to first inappropriate therapy – VVI vs. VDD

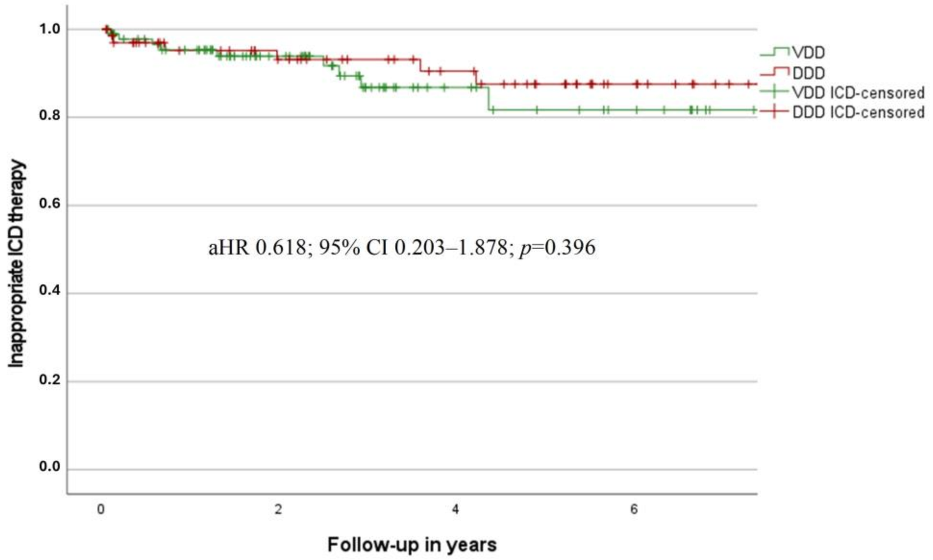

Patients at risk	1 year	2 years	3 years	4 years	5 years	6 years	7 years	8 years
**VDD**	94	75	52	30	19	14	11	1
**DDD**	69	52	45	38	32	24	15	8

Figure S2D Time to first inappropriate therapy – VDD vs. DDD

Table S12. Multivariate analysis of time to first hospitalization due to arrhythmic cause **- VVI vs. VDD**

Risk factor	unadjusted HR (95% CI)	p-value	adjusted HR (95% CI)	p-value
ICD type	1.463 (0.899-2.379)	**0.125**	1.706 (1.043-2.792)	**0.033**
Age	1.009 (0.988-1.031)	0.390		
Male	0.733 (0.430-1.248)	0.253		
Primary prophylaxis	2.804 (1.339-5.869)	**0.006**	3.041 (1.443-6.411)	**0.003**
Ischemic etiology	0.770 (0.475-1.248)	0.289		
Previously diagnosed atrial fibrillation	0.981 (0.576-1.671)	0.943		
Hypertension	0.939 (0.376-2.348)	0.893		
Dyslipidaemia	1.410 (0.672-2.957)	0.363		
Diabetes mellitus	1.380 (0.840-2.267)	0.203		
Stroke/TIA	0.881 (0.353-2.198)	0.786		
Bradypacing indication	1.258 (0.174-9.096)	0.820		
LVEF	0.995 (0.977-1.013)	0.555		
QRS width	0.995 (0.981-1.009)	0.491		
Heart rate	0.991 (0.973-1.009)	0.328		
Creatinine	1.004 (0.999-1.008)	0.104		
Hemoglobin	0.996 (0.982-1.011)	0.620		
Remote monitoring	1.394 (0.848-2.289)	0.190		
Antiplatelet therapy	1.054 (0.639-1.737)	0.838		
Anticoagulation	0.760 (0.459-1.260)	0.288		
Beta-blockers	0.806 (0.197-3.300)	0.764		
ACEI/ARB/ARNI	0.415 (0.226-0.761)	**0.004**	0.404 (0.219-0.745)	**0.004**
Diuretics	0.939 (0.581-1.518)	0.797		
Calcium channel blockers	1.555 (0.905-2.672)	0.110		
Mineralocorticoid receptor antagonists	0.803 (0.495-1.302)	0.374		
Statins	0.867 (0.520-1.444)	0.583		
Amiodarone	1.618 (0.942-2.778)	**0.081**	1.277 (0.734-2.220)	0.387
Digitalis glycosides	0.619 (0.225-1.703)	0.353		

Table S13. Multivariate analysis of time to first hospitalization due to arrhythmic cause **- VDD vs. DDD**

Risk factor	unadjusted HR (95% CI)	p-value	adjusted HR (95% CI)	p-value
ICD type	0.638 (0.366-1.113)	**0.114**	0.700 (0.365-1.341)	0.282
Age	1.009 (0.987-1.030)	0.429		
Male	0.960 (0.523-1.761)	0.895		
Primary prophylaxis	1.523 (0.830-2.796)	0.175		
Ischemic etiology	0.936 (0.548-1.601)	0.810		
Previously diagnosed atrial fibrillation	1.106 (0.628-1.949)	0.727		
Hypertension	1.694 (0.527-5.439)	0.376		
Dyslipidaemia	1.117 (0.561-2.221)	0.753		
Diabetes mellitus	1.221 (0.709-2.103)	0.472		
Stroke/TIA	0.643 (0.231-1.785)	0.396		
Bradypacing indication	0.647 (0.332-1.261)	0.201		
LVEF	0.986 (0.969-1.004)	0.117		
QRS width	1.002 (0.990-1.014)	0.786		
Heart rate	0.983 (0.962-1.003)	0.101		
Creatinine	1.005 (1.000-1.009)	**0.041**	1.005 (1.000-1.009)	**0.049**
Hemoglobin	1.005 (0.988-1.022)	0.594		
Remote monitoring	1.466 (0.862-2.492)	0.158		
Antiplatelet therapy	1.012 (0.586-1.746)	0.967		
Anticoagulation	1.118 (0.651-1.920)	0.686		
Beta-blockers	1.464 (0.525-4.086)	0.466		
ACEI/ARB/ARNI	0.764 (0.384-1.519)	0.442		
Diuretics	1.199 (0.706-2.037)	0.501		
Calcium channel blockers	1.748 (1.001-3.051)	**0.050**	1.412 (0.770-2.589)	0.265
Mineralocorticoid receptor antagonists	0.710 (0.398-1.265)	0.245		
Statins	0.788 (0.451-1.379)	0.404		
Amiodarone	2.744 (1.513-4.976)	**0.001**	2.761 (1.461-5.218)	**0.002**
Digitalis glycosides	0.885 (0.215-3.640)	0.865		

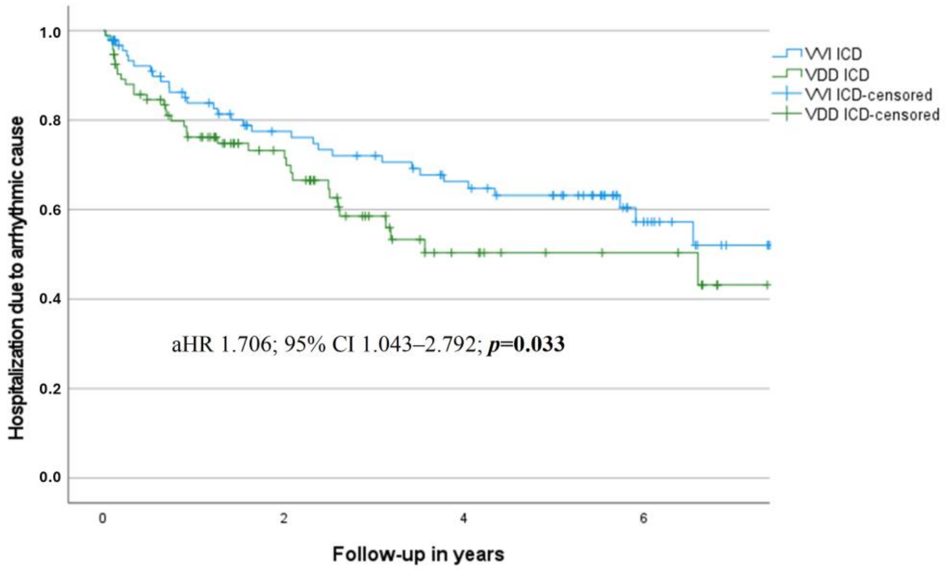

Patients at risk	1 year	2 years	3 years	4 years	5 years	6 years	7 years	8 years
**VVI**	93	69	57	52	44	37	16	6
**VDD**	94	62	44	24	14	9	8	1

Figure S3A Time to first hospitalization due to arrhythmic cause – VVI vs. VDD

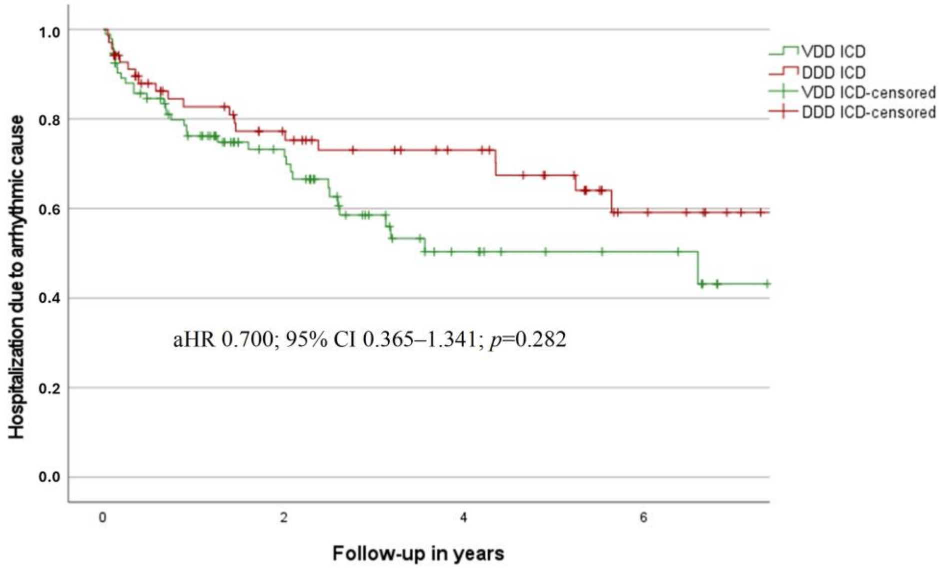

Patients at risk	1 year	2 years	3 years	4 years	5 years	6 years	7 years	8 years
**VDD**	94	62	44	24	14	9	8	1
**DDD**	69	47	39	32	28	21	10	5

Figure S3B Time to first hospitalization due to arrhythmic cause – VDD vs. DDD

Table S14. Arrhythmia-related hospitalization events in the different groups

	**VVI (N=93)**	**VDD (N=94)**	**DDD (N=69)**
Total number of first arrhythmia-related hospitalization events	32	35	21
Admission due to ventricular tachycardia or electric storm (n, %)	27 (84%)	23 (66%)	10 (48%)
Admission due to management of supraventricular tachycardia (including elective admission due to ECV or catheter ablation) (n, %)	3 (10%)	8 (23%)	5 (24%)
Admission due to other causes (electrode malfunction, BIV-upgrade, syncope, etc.)	2 (6%)	4 (11%)	6 (28%)

Table S15. Multivariate analysis of time to first hospitalization due to heart failure **- VVI vs. VDD**

**Risk factor**	**unadjusted HR (95% CI)**	**p-value**	**adjusted HR (95% CI)**	**p-value**
ICD type	0.949 (0.449-2.006)	**0.891**	1.628 (0.619-4.279)	0.323
Age	0.989 (0.959-1.020)	0.477		
Male	0.670 (0.305-1.474)	0.320		
Primary prophylaxis	0.330 (0.159-0.685)	**0.003**	0.399 (0.136-1.170)	0.094
Ischemic etiology	0.809 (0.389-1.681)	0.570		
Previously diagnosed atrial fibrillation	2.329 (1.114-4.867)	**0.025**	2.160 (0.670-6.967)	0.197
Hypertension	0.660 (0.198-2.197)	0.499		
Dyslipidaemia	1.584 (0.478-5.252)	0.452		
Diabetes mellitus	1.712 (0.816-3.590)	0.155		
Stroke/TIA	2.544 (0.959-6.745)	**0.061**	1.980 (0.628-6.240)	0.243
Bradypacing indication	0.048 (0.000-52297.115)	0.669		
LVEF	0.957 (0.925-0.990)	**0.011**	1.002 (0.961-1.045)	0.917
QRS width	1.005 (0.985-1.025)	0.645		
Heart rate	1.021 (1.000-1.042)	**0.046**	1.023 (0.997-1.051)	0.087
Creatinine	1.010 (1.003-1.017)	**0.005**	1.012 (1.004-1.020)	**0.003**
Hemoglobin	0.973 (0.954-0.993)	**0.009**	0.958 (0.937-0.980)	**<0.001**
Remote monitoring	0.697 (0.297-1.635)	0.407		
Antiplatelet therapy	0.692 (0.333-1.439)	0.324		
Anticoagulation	1.875 (0.900-3.908)	**0.093**	0.649 (0.239-1.764)	0.397
Beta-blockers	21.124 (0.002-267352.000)	0.527		
ACEI/ARB/ARNI	0.501 (0.191-1.314)	0.160		
Diuretics	2.801 (1.237-6.339)	**0.013**	1.378 (0.460-4.130)	0.567
Calcium channel blockers	0.414 (0.125-1.368)	0.414		
Mineralocorticoid receptor antagonists	3.849 (1.561-9.489)	**0.003**	2.827 (0.934-8.560)	0.066
Statins	0.566 (0.270-1.186)	0.132		

Amiodarone	0.719 (0.250-2.067)	0.719		
Digitalis glycosides	4.770 (2.097-10.848)	**<0.001**	3.521 (1.224-10.132)	**0.020**

Table S16. Multivariate analysis of time to first hospitalization due to heart failure **- VDD vs. DDD**

Risk factor	unadjusted HR (95% CI)	p-value	adjusted HR (95% CI)	p-value
ICD type	0.586 (0.219-1.570)	**0.287**	0.949 (0.301-2.991)	0.928
Age	0.987 (0.957-1.019)	0.436		
Male	0.633 (0.237-1.689)	0.362		
Primary prophylaxis	0.379 (0.150-0.962)	**0.041**	0.413 (0.151-1.126)	0.084
Ischemic etiology	0.870 (0.343-2.206)	0.769		
Previously diagnosed atrial fibrillation	1.556 (0.596-4.060)	0.366		
Hypertension	0.757 (0.172-3.326)	0.712		
Dyslipidaemia	0.858 (0.280-2.627)	0.789		
Diabetes mellitus	1.042 (0.391-2.781)	0.934		
Stroke/TIA	1.141 (0.262-4.971)	0.860		
Bradypacing indication	0.817 (0.268-2.487)	0.722		
LVEF	0.941 (0.903-0.980)	**0.003**	0.957 (0.922-0.993)	**0.019**
QRS width	0.999 (0.977-1.021)	0.895		
Heart rate	1.002 (0.972-1.033)	0.887		
Creatinine	1.010 (1.003-1.018)	**0.003**	1.011 (1.003-1.018)	**0.005**
Hemoglobin	0.984 (0.958-1.011)	0.244		
Remote monitoring	0.812 (0.314-2.101)	0.668		
Antiplatelet therapy	0.736 (0.290-1.867)	0.519		
Anticoagulation	1.010 (0.389-2.622)	0.983		
Beta-blockers	23.676 (0.024-23075.647)	0.367		
ACEI/ARB/ARNI	0.635 (0.208-1.933)	0.423		
Diuretics	1.744 (0.676-4.501)	0.250		
Calcium channel blockers	0.364 (0.084-1.584)	0.178		
Mineralocorticoid receptor antagonists	1.901 (0.746-4.840)	0.178		
Statins	0.666 (0.258-1.719)	0.401		
Amiodarone	1.079 (0.310-3.762)	0.904		
Digitalis glycosides	1.572 (0.209-11.839)	0.661		

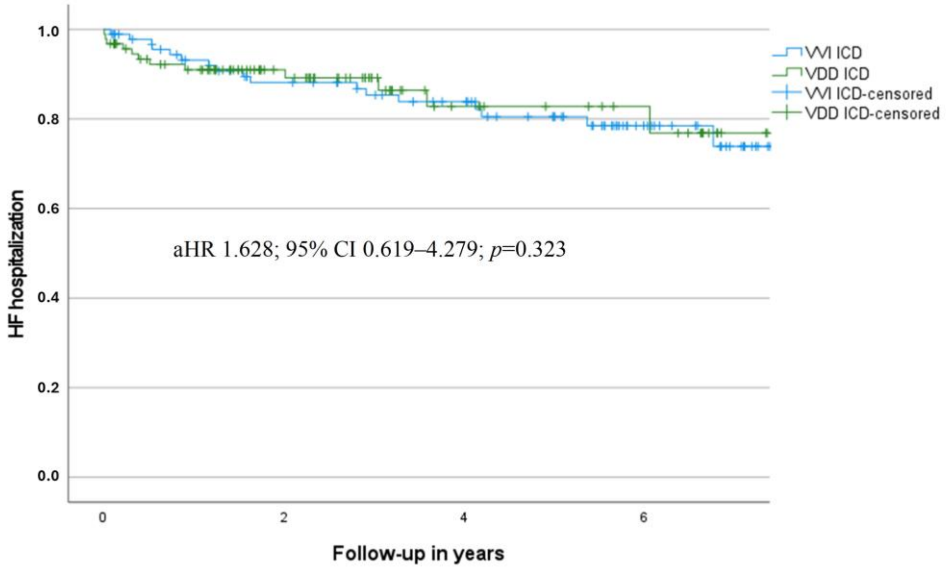

Patients at risk	1 year	2 years	3 years	4 years	5 years	6 years	7 years	8 years
**VVI**	93	77	67	60	54	43	24	12
**VDD**	94	74	51	33	21	17	14	2

Figure S4A Time to first heart failure (HF) hospitalization – VVI vs. VDD

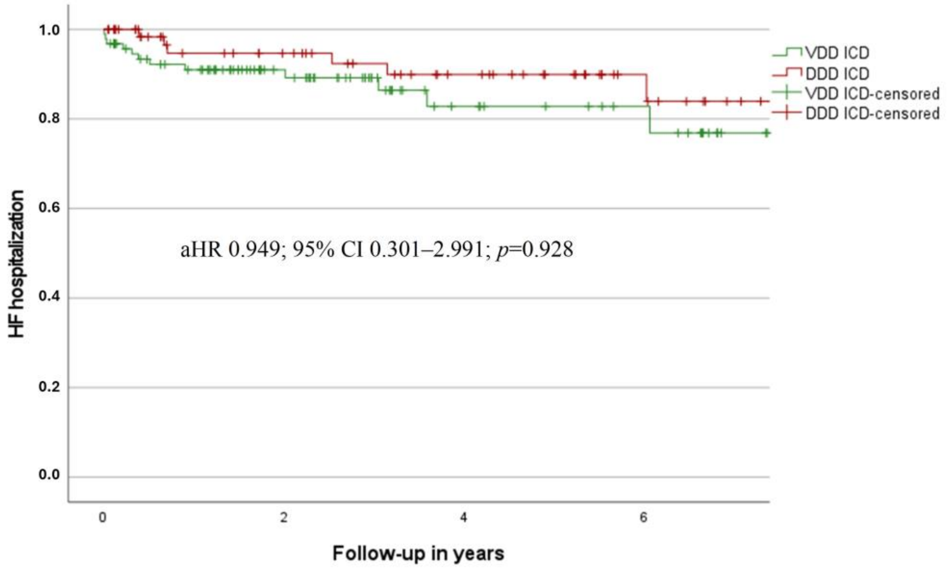

Patients at risk	1 year	2 years	3 years	4 years	5 years	6 years	7 years	8 years
**VDD**	94	74	51	33	21	17	14	2
**DDD**	69	50	45	38	31	24	15	8

Figure S4B Time to first HF hospitalization – VDD vs. DDD

Table S17. Multivariate analysis of all-cause mortality **- all groups**

Risk factor	unadjusted HR (95% CI)	p-value	adjusted HR (95% CI)	p-value
ICD type	0.906 (0.696-1.179)	**0.463**	0.960 (0.711-1.295)	0.787
Age	1.060 (1.037-1.084)	**<0.001**	1.060 (1.031-1.089)	**<0.001**
Male	0.883 (0.546-1.426)	0.611		
Primary prophylaxis	0.849 (0.534-1.349)	0.488		
Ischemic etiology	1.297 (0.830-2.028)	0.253		
Previously diagnosed atrial fibrillation	1.958 (1.268-3.023)	**0.002**	1.536 (0.936-2.521)	0.089
Hypertension	4.969 (1.220-20.233)	**0.025**	1.177 (0.272-5.094)	0.828
Dyslipidaemia	1.645 (0.872-3.105)	0.125		
Diabetes mellitus	1.968 (1.277-3.033)	**0.002**	2.215 (1.361-3.607)	**0.001**
Stroke/TIA	1.409 (0.726-2.733)	0.311		
Bradypacing indication	1.035 (0.575-1.864)	0.908		
LVEF	0.975 (0.959-0.991)	**0.002**	0.967 (0.948-0.986)	**0.001**
QRS width	1.008 (0.997-1.018)	0.146		
Heart rate	1.017 (1.003-1.030)	**0.013**	1.005 (0.992-1.018)	0.460
Creatinine	1.002 (0.997-1.006)	0.486		
Hemoglobin	0.990 (0.977-1.004)	0.163		
Remote monitoring	0.248 (0.114-0.538)	**<0.001**	0.320 (0.145-0.709)	**0.005**
Antiplatelet therapy	0.948 (0.603-1.489)	0.816		
Anticoagulation	1.840 (1.190-2.843)	**0.006**	1.172 (0.637-2.158)	0.609
Beta-blockers	0.935 (0.407-2.152)	0.875		
ACEI/ARB/ARNI	1.023 (0.554-1.889)	0.942		
Diuretics	1.740 (1.111-2.725)	**0.015**	1.451 (0.855-2.463)	0.167
Calcium channel blockers	0.888 (0.519-1.519)	0.666		
Mineralocorticoid receptor antagonists	1.347 (0.870-2.085)	0.182		
Statins	1.114 (0.686-1.808)	0.662		
Amiodarone	1.244 (0.729-2.125)	0.423		
Digitalis glycosides	1.021 (0.470-2.220)	0.958		

Table S18. Distribution of structural heart diseases between all groups

	VVI (N=93)	VDD (N=94)	DDD (N=69)
Ischemic cardiomyopathy (n,%)	56 (60%)	58 (62%)	39 (57%)
Dilatative cardiomyopathy (n,%)	30 (32%)	26 (28%)	9 (13%)
Hypertrophic cardiomyopathy (n,%)	0 (0%)	4 (4%)	9 (13%)
Arrhythmogenic right ventricular cardiomyopathy (n,%)	0 (0%)	0 (0%)	1 (2%)
Long QT syndrome (n,%)	1 (1%)	0 (0%)	4 (6%)
Ischemic cardiomyopathy (n,%)	56 (60%)	58 (62%)	39 (57%)
Dilatative cardiomyopathy (n,%)	30 (32%)	26 (28%)	9 (13%)

Disclaimer/Publisher's Note: The statements, opinions and data contained in all publications are solely those of the individual author(s) and contributor(s) and not of MDPI and/or the editor(s). MDPI and/or the editor(s) disclaim responsibility for any injury to people or property resulting from any ideas, methods, instructions or products referred to in the content.
